# Rapid climate change results in long-lasting spatial homogenization of phylogenetic diversity

**DOI:** 10.1038/s41467-020-18343-6

**Published:** 2020-09-16

**Authors:** Bianca Saladin, Loïc Pellissier, Catherine H. Graham, Michael P. Nobis, Nicolas Salamin, Niklaus E. Zimmermann

**Affiliations:** 1 Swiss Federal Research Institute WSL, 8903 Birmensdorf, Switzerland; 2 Department of Environmental Systems Sciences, Landscape Ecology, Institute of Terrestrial Ecosystems, ETH Zürich, 8092 Zurich, Switzerland; 3 Department of Computational Biology, University of Lausanne, 1015 Lausanne, Switzerland

**Keywords:** Biodiversity, Climate-change ecology, Plant evolution, Biogeography

## Abstract

Scientific understanding of biodiversity dynamics, resulting from past climate oscillations and projections of future changes in biodiversity, has advanced over the past decade. Little is known about how these responses, past or future, are spatially connected. Analyzing the spatial variability in biodiversity provides insight into how climate change affects the accumulation of diversity across space. Here, we evaluate the spatial variation of phylogenetic diversity of European seed plants among neighboring sites and assess the effects of past rapid climate changes during the Quaternary on these patterns. Our work shows a marked homogenization in phylogenetic diversity across Central and Northern Europe linked to high climate change velocity and large distances to refugia. Our results suggest that the future projected loss in evolutionary heritage may be even more dramatic, as homogenization in response to rapid climate change has occurred among sites across large landscapes, leaving a legacy that has lasted for millennia.

## Abstract (web summary)

How past climate change has affected biodiversity over large spatial scales remains underexplored. Here, the authors find marked homogenization in flowering plant phylogenetic diversity across Central and Northern Europe linked to rapid climate change and large distances to glacial refugia.

## Introduction

Climate stability through geological time allows more species to evolve and persist, ultimately resulting in higher biodiversity^1–3^. Climatically stable regions host endemic and small ranged size species^2^ leading to high turnover of species along spatial and environmental gradients, and high turnover of lineages when considering their phylogenetic relatedness^4^. In contrast, rapid changes in climate have been shown to strongly affect biodiversity patterns by selecting against endemic species, and instead favoring species with strong dispersal capabilities which results in the persistence of generalist species with large range sizes^5,6^. Today, ecosystems are exposed to rapid climate change, forcing species to readjust their ranges and to track suitable habitats^7–11^. Such high climate change velocity will potentially leave a marked imprint on the future biogeography of clades^12^. By projecting phylogenetic diversity through time, an increasing number of studies warn of a significant loss of evolutionary heritage from climate change and call for conservation of global genetic diversity^12–15^. While these studies analyzed temporal patterns of phylogenetic diversity at given sites, little is known about the imprints of fast climate change on the spatial variation in phylogenetic diversity (phylogenetic turnover, phylo-*β* hereafter) among sites across large regions. Spatial phylo-*β* provides information on how much diversity a landscape can maintain and where in geographic space lineage diversity varies due to past region-specific processes^16^. Understanding the effects of past rapid climatic changes on spatial phylo-*β* is crucial for anticipating the dramatic effects of ongoing climate changes on future species distributions and extinctions^17,18^.

Biodiversity patterns emerge from ecological and evolutionary processes including immigration, competition, speciation, and extinction^19^. These processes are lineage-specific because lineages vary in their dispersal ability and capacity to adapt to certain environments, leading to turnover of clades across environmental^20^ and geographic^21^ space. Landscapes of high environmental diversity offer more niche space for adaptation, resulting in faster lineage diversification. Topographically complex regions are characterized by natural migration barriers and enhance allopatric speciation^19^. Disturbances such as glacial cycles can reset and reduce the diversity^22,23^. All these processes affect spatial patterns in phylo-*β* and are mediated by the rate and extent of climate change.

Phylo-*β* increases with geographic and environmental distance^20^ and with richness difference^24^ among sites. To assess the effect of climate change on phylo-*β* across large landscapes, one has to remove the effect of richness differences (using Simpson based phylo-*β*^24^) and the effect of geographic and environmental distances among sites (corrected, Simpson-based phylo-*β*^20^; phylo-*β*_simC_ hereafter). This makes comparisons across regions independent of local topographic complexity.

Here, we quantify the phylo-*β*_simC_ of seed plants across Europe among neighboring cells. Europe is ideal for analyzing the effects of rapid climate change on phylo-*β*_simC_, as climate oscillations and glaciations have strongly influenced current species distributions^25,26^. European species distributions have expanded and contracted through history resulting in both extinctions^27–29^, and species persistence in small, scattered southern refugia during cold periods. From there, they expanded northwards or to higher elevations during subsequent warmings^27,30–32^. Some species might also have survived in northern refugia, although this is debated^33,34^. These processes likely strongly influenced species compositions in regions of high climatic oscillations and away from refugia areas by resetting the northern diversity. We expect that these historical changes left a strong imprint on phylogenetic structure across regions. More specifically, we expect lower phylo-*β*_simC_ (i) in regions of high past climate change velocity, (ii) in regions with increasing distance to refugia, (iii) and where range sizes of species are larger. To test these hypotheses, we evaluated the relative importance of past climate change velocity^35^, distance to refugia using dynamic range expansion simulations^36^ and mean range size of the local species pool^20^ in explaining phylo-*β*_simC_. We analyze phylo-*β*_simC_ separately for angiosperms and gymnosperms.

Our results reveal a strong spatial homogenization in phylogenetic seed plant diversity across Central and Northern Europe. These patterns emerge when analyzing phylogenetic turnover among neighboring cells that is corrected for environmental and geographic distance effects. The resulting pattern is best explained by rapid Quaternary climate change and distance to glacial refugia, indicating that only a subset of species with suitable conditions farther north has tracked the warming since LGM from the southern refugia. The coincidence of hypothesized northern refugia with regions of elevated spatial turnover in Central and Northern Europe in our results supports the idea that refugia existed in regions north of the southern mountain systems.

## Results

### Spatial patterns in phylogenetic turnover across Europe

**Fig. 1:**
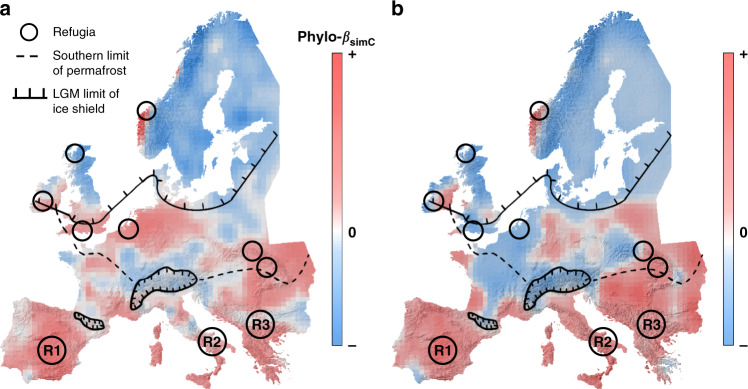
Phylo-*β*_simC_ across Europe. **a, b** Phylo-*β*_simC_ is defined as the residuals of each raster cell to its 24 nearest neighbors from a generalized linear model with true phylogenetic turnover (phylo-*β*_sim_, corrected for species richness effects) as dependent variable and environmental and geographic distance among sampled sites as explanatory variables for angiosperms (**a**) and gymnosperms (**b**). Circles indicate refugia with the three main potential refugia in the South (R1: Portugal–Spain, R2: Italy, and R3: Balkans) and potential Northern refugia redrawn from refs. ^27,52^ indicated by smaller, unlabeled circles. The southern limit of the permafrost and the maximum extension of ice sheets in Europe during the last glacial maximum are illustrated by the hashed line and the scaled line, respectively, with the ice on the scaled side of the line (redrawn from Taberlet and colleagues^27^). The underlying hillshade maps of Europe are based on the digital elevation model of the European Environment Agency^81^.

In agreement with our general expectation, phylo-*β*_simC_ (i.e., Simpson’s phylo-*β* corrected for local environmental or geographic distances) of both angiosperms and gymnosperms was strongly structured across Europe and generally higher in Southern than in Northern Europe, with some peaks and troughs in Central Europe (Fig. 1 and Supplementary Fig. 1). In angiosperms, low phylo-*β*_simC_ was not only found in Northern Europe, but also within, north and east of the Alps. In contrast, high phylo-*β*_simC_ was generally found in Southern Europe, but also in the Carpathians, Benelux, Northern Germany, and South England. A similar pattern was found for gymnosperms, but with low phylo-*β*_simC_ for most of the Northern Atlantic coast and the Tatra mountains (north-western Carpathians).

### Drivers of spatial phylogenetic turnover across Europe

**Fig. 2:**
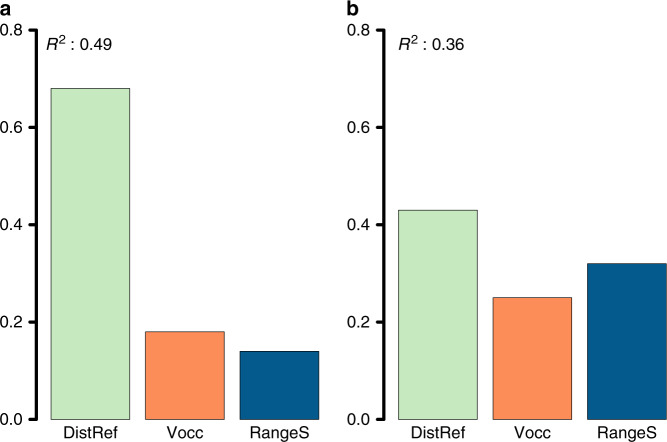
Relative variable importance on phylo-β_simC_. **a, b** Variable importance of distance to refugia (DistRef), velocity of climate change (Vocc) and range size (RangeS), in explaining phylo-*β*_simC_ for angiosperms (**a**) and gymnosperms (**b**). *R*^2^ represents the model fit of a standardized linear regression to explain phylo-*β*_simC_ by means of DistRef, Vocc, and RangeS, (linear and quadratic terms). Relative variable importance was rescaled to sum to 1.0.

**Fig. 3:**
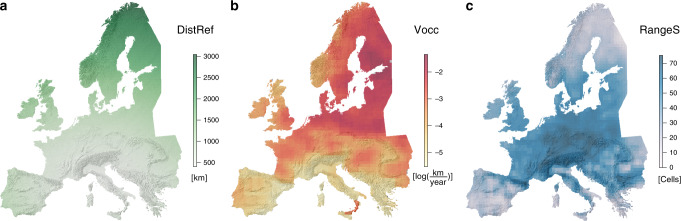
Explanatory variables of angiosperm phylo-β_simC_. **a** Mean distance to refugia (DistRef) of angiosperms was calculated as the average distance among all species per cell to its reconstructed LGM distribution limits (see Fig. 4 for species’ LGM distributions). **b** The velocity of climate change (Vocc) since the LGM was calculated based on 1000-year time steps of reconstructed temperature and precipitation trends since the LGM. Vocc considers both the rate of climate change and the rate of elevation change of the local topography and thus expresses the average spatial change of climatic variables over time from the LGM to the present. **c** Mean range size (RangeS) of the local species pool of angiosperms that was calculated as the average range size of all species per cell across their European distribution as mapped in the Atlas Florae Europaeae. The underlying hillshade maps of Europe are based on the digital elevation model of the European Environment Agency^81^.

**Fig. 4:**
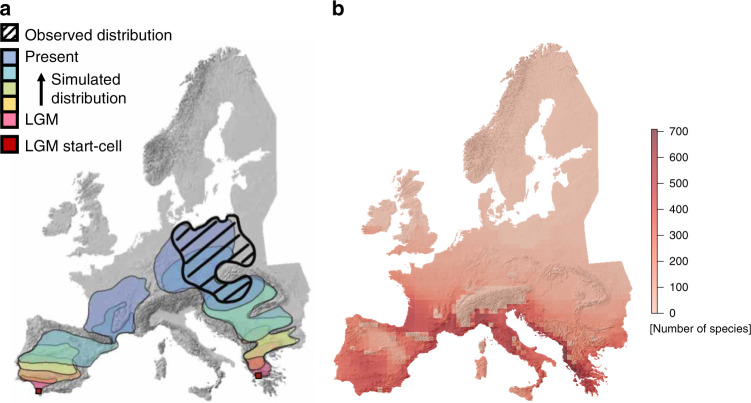
Dynamic range expansion since LGM and angiosperm diversity during LGM. **a** Illustration of dynamic range expansion from two possible start-cells during the last glacial maximum (LGM) using KISSMig^36^ that had suitable LGM climate for a given species. Dynamic simulations from all cells that had suitable climates during LGM were used for each species to identify LGM refugial cells from which current distribution ranges likely were colonized. Colonization from SW Europe does not represent a likely colonization of the current habitat (and is rejected as refugial cell) while colonization from SE Europe does. **b** Angiosperm richness of identified LGM refugia across all species using dynamic KISSMig simulations. The underlying hillshade maps of Europe are based on the digital elevation model of the European Environment Agency^81^.

The phylo-*β*_simC_ pattern of angiosperms (Fig. 2a) were well explained (*R*^2^ = 0.49) by the three explanatory variables (Fig. 3): mean distance to the LGM refugia (DistRef), past climate change velocity (Vocc), and mean range size of species assemblages (RangeS). The relative contribution of the three variables to the total model fit was 68% (DistRef), 18% (Vocc), and 14% (RangeS). A different result emerged for gymnosperms (Fig. 2b and Supplementary Fig. 2). The explanatory model reached a lower calibration strength (*R*^2^ = 0.36), and the ranking of the relative contribution of the three variables changed with 43% (DistRef), 25% (Vocc), and 32% (RangeS). Similar explanatory results were found (Supplementary Fig. 3) when climate stability since LGM (ClimStab) was used instead of DistRef, which are highly correlated (*r* = −0.77 in angiosperms, *r* = −0.79 in gymnosperms). Southern Europe is characterized by a high species diversity of seed plants that persisted in refugia during the LGM (Fig. 4 and Supplementary Fig. 4). The patterns in phylo-*β*_simC_ were negatively related to distances to LGM refugia (Fig. 3 and Supplementary Fig. 2). While past climate change velocities were negatively related to phylo-*β*_simC_, climate stability and species range size were positively related to phylo-*β*_simC_ (Fig. 3 and Supplementary Fig. 2).

### Local distance effects of geography and environment

Environmental and geographic distances regressed positively (adjusted regression *R*^2^ for angiosperms: 0.31, gymnosperms: 0.13) with true phylogenetic turnover (phylo-*β* corrected for species richness differences; Supplementary Fig. 5). The removal of these distance effects among neighboring sites on true phylogenetic turnover, to obtain phylo-*β*_simC_, was strongest in mountains and along coasts (Supplementary Fig. 6).

## Discussion

In agreement with our general expectation, we find a clear spatial structure in phylogenetic turnover of seed plants across Europe, independent of regional environmental or topographic heterogeneity (phylo-*β*_simC_). Past climate change appears to strongly influence this pattern. Congruent with our first hypothesis, we find higher phylo-*β*_simC_ in regions of lower past climate change velocity (mostly Southern Europe) and lower phylo-*β*_simC_ in Northern Europe, which was exposed to high velocity and instability in climate. This indicates a marked regional homogenization of the flora (low phylo-*β*_simC_) in response to rapid climatic changes since LGM in Northern Europe. Previous studies have forecasted a loss in evolutionary heritage through time due to rapid future climate change^13,37^, indicating local homogenization. Our results indicate that the loss may even be more dramatic, as homogenization also might occur among local sites across large landscapes.

Different mechanisms can explain low phylo-*β*_simC_ in regions of high climate change velocity. First, our results are in line with previous findings on the degree of range filling for European plants^38–40^. These results indicate that many European plant species have not yet fully recolonized all suitable sites in Central and Northern Europe. Further, central and northern regions have been colonized by rather closely related generalist species with rapid dispersal capacities and, consequently, large ranges, while specialists generally have slower migration capacities and, thus, more restricted ranges^41^. Many of these specialists have not been able to migrate to these more distant regions that became ice-free around 10 millennia ago. Spatial turnover is thus linked to range size with smaller ranges enhancing turnover^42^. Second, wide-spread species exhibit larger niche breadths both in animals^43^ and plants^43,44^. These widespread generalists are more likely to survive during climatic shifts and are less prone to extinction^5,43,45^. Moreover, regions of rapid historical climatic shifts have been associated with reduced local diversity, a marked absence of small ranged or endemic amphibians, birds and mammals^4,6^, and a proliferation of large ranged generalists^5^. A similar result was found for European dung beetles, with phylogenetic clustering of generalists with large ranges for Northern Europe and diverse variable range sizes and life strategies in Southern Europe^46^, although no correction for regional heterogeneity effects was considered. If phylogenetically clustered, the discussed mechanisms lead to low phylo-*β*_simC_ as found in our study and supporting our third hypothesis of lower phylo-*β*_simC_, where range sizes of species are larger.

Regions of high phylo-*β*_simC_ are candidates for quaternary refugia. In agreement with our second hypothesis, we found a negative relationship between distance to refugia and phylo-*β*_simC_, especially in angiosperms. Three main refugia for different biota in Southern Europe have previously been documented (Iberian–Peninsula, Italy, and in the Balkans, Fig. 1^27,31^) and their positions agree with our patterns of both low distance to refugia and high phylo-*β*_simC_. The postglacial recolonization dynamics across Europe were diverse^47^ and likely not all species have expanded sufficiently to fully fill their potential ranges in response to rapid postglacial warming^38,48^. As a result, and confirming the importance of historical processes, the contemporary distribution of many small ranged (southern) species has been best explained by distance to refugia rather than current climate^41^. These findings support our result of the importance of historical processes compared to those of contemporary drivers such as environmental or geographic distance (Supplementary Fig. 7).

In Northern Europe species assemblages seem to be phylogenetically clustered, characterized by low phylo-*β*_simC_. However, we find some locations of high phylo-*β*_simC_ in Central and Northern Europe, away from the well-known Southern refugia. Northern refugia had been proposed, especially for cold-adapted species^49–55^, although this remains controversial^33,34^. The higher phylo-*β*_simC_ in Central and Northern Europe largely matches the proposed locations of these hypothesized northern refugia (Fig. 1), giving support to the idea of persistent northern refugia during the Ice Age. Such refugia may have originated from locally more diverse environments, leading to a greater likelihood of refugial survival for some species in these heterogenous areas. Alternatively, higher phylo-*β*_simC_ in these refugial areas may simply arise from a lack of migration away from quaternary refugia after climate warming, now leading to higher spatial turnover.

Our results are robust regarding the use of different phylogenetic sources (Supplementary Fig. 8), and for both gymnosperms and angiosperms. The fact that both gymnosperms and angiosperms showed very similar results suggests that the angiosperm results may be robust irrespective of analyzing only the currently available 25% of European angiosperm species. This consistency in our results suggests that the same mechanisms likely drove these broad-scale biodiversity patterns across clades of life in Europe. Further, the analysis scale of 50 × 50 km in combination with removing local topographic effects on phylo-*β*_simC_ has proven to be appropriate for our analyses. Had we analyzed finer-scale data, local topographic and environmental effects might have been too dominant to discover larger-scale legacy effects (but see ref. ^56^).

In summary, our results demonstrate the clear biodiversity imprint of the Quaternary climate oscillations on spatial phylogenetic beta diversity which has left a legacy on current biodiversity patterns. Projected future climate change is of equal, if not greater intensity^57^, and may leave a strong imprint on the global phylogenetic diversity. Although logical, it is alarming that the projected centers of loss in suitable plant habitats in previous studies^37^ match areas of high regional phylo-*β*_simC_. This points to an additional loss in regional diversity through spatial homogenization processes as discussed above compared to a projected local diversity loss through time. We show that the effect of climate changes on biodiversity has lasted through millennia and the same can be expected of on-going and future changes.

## Methods

### Extent of study area

The study area spans the entire European subcontinent, but some smaller and less connected islands (Iceland, Balearics, and Malta) and some eastern parts of Europe were removed due to a lack of quality in occurrence data in those regions (Belarus, Russia, Ukraine, and Turkey).

### Distribution data

Seed plant distribution data of angiosperms and gymnosperms were taken from the digital maps of the *Atlas Florae Europaeae* (AFE^58,59^) which includes ca. 25% of all European vascular plants. AFE maps are available for all European gymnosperms, while only ca. 25% of the angiosperms are mapped to date. The phylogenetic position of the available AFE angiosperm and gymnosperm data is illustrated in Supplementary Fig. 9. This atlas provides distribution data for each species mapped as presence or absence for each of quasi-rectangular polygons mimicking a 50 × 50 km raster across Europe (see Supplementary Fig. 1). We extracted plant records within the extent of our study area, totaling to 1970 sample points representing centroids of the AFE distribution polygons (AFE points hereafter). Using these data, we generated community data of all species of gymnosperms and angiosperms present at these AFE points. The database includes native and naturalized alien taxa from Europe of which we only included plant records of native taxa. To maximize the degree of matching between the species names of the available distribution data and those from the phylogenetic trees, we checked and standardized the species names according to *The Plant List*^60^ (TPL) using the *Taxonstand*^61^ package in the R environment^62^, as recommended by Qian and Jin^63^. We replaced species names considered as synonyms in TPL with their accepted names according to TPL and corrected typos in species names. Intraspecific taxa were combined with their parental species. We checked species names not found in TPL manually within the *Euro Plus Med Plantbase*^64^ (E + M). According to this, we kept five species (*Cotoneaster majoricensis, Malus crescimannoi, Papaver ernesti-mayeri, Pyrus castribonensis*, and *Pyrus sicanorum*) in our data as their names are accepted in E + M. Further, we did not change the names of genera where E + M suggested names different from those in TPL (e.g., *Alyssum nebrodense* is *Odontarrhena nebrodensis* in E + M), nor did we change names where another synonym represents the accepted name (e.g., *Iberis boppardiensis subsp. stricta* would be *I. linifolia subsp. stricta*). For subspecies that were not found in TPL, we changed the subspecies names to the species parental names following E + M (e.g., *Cardamine raphanifolia subsp. barbareoides* was changed to *Cardamine barbareoides*). All subspecies of *Ranunculus auricomus* were conservatively merged to *Ranunculus auricomus*, although E + M lists them as accepted species. Finally, we split the retained data into the two major clades, angiosperms and gymnosperms, and removed all other vascular plants. The angiosperm data set consists of 4003 species, 319 genera and 42 families, and the gymnosperm data set includes 41 species, 9 genera and 4 families. The cleaned distribution data were available as a point database with somewhat irregularly spaced sample points in lon/lat coordinates and an average distance among points of ca. 50 km. To calculate true distances, we re-projected the point coordinates to a metric projection, namely the Lambert azimuthal equal-area projection (LAEA).

### Phylogenetic data

We used the species-level plant megaphylogeny (PhytoPhylo) of Qian and Jin^63^ as a backbone tree and phylogenies of Smith and Brown^65^ to test for robustness of our results (Supplementary Fig. 8). The PhytoPhylo megaphylogeny is an updated species-level phylogeny of the earlier published species-level phylogeny by Zanne et al.^66^, which is based on sequences of seven gene regions available in GenBank^66^ (18S rDNA, 26S rDNA, ITS, *matK*, *rbcL*, *atpB*, and *trnL-F*). PhytoPhylo includes all families of extant seed plants in the world^67^. We first checked for concordance in names between tips of the phylogenetic tree and species which were included in the AFE data. While 51 angiosperm genera were not found in the PhytoPhylo tree, all gymnosperm genera were included. We removed the 99 species from the AFE data belonging to the 51 angiosperm genera not available in PhytoPhylo (see Supplementary Table 1 for the full lists) because they could not be unambiguously assigned to a synonym in the phylogenetic trees (see Supplementary Fig. 10 for results when including the species belonging to those genera by adding them as basal polytomies within the corresponding family). This resulted in a total of 3904 angiosperm species, 268 genera, and 41 families in the AFE data. We then generated the species level phylogenies for the adjusted AFE species list based on the species-level PhytoPhylo megaphylogeny^63^ using the S.PhyloMaker package^63^. S.PhyloMaker was used to link the AFE plant names with those in PhytoPhylo and to prune off tips that were not included in the AFE data. For names in the AFE dataset not found in PhytoPhylo, we used SPhyloMaker to add them to the megaphylogeny within the respective genera. This can be done using three different approaches, adding: (A1) genera or species as basal polytomies within their families or genera, (A2) genera or species randomly within their families or genera, and (A3) genera or species as polytomies within their parental taxa using the same approach as Phylomatic and assigning branch lengths using the function BLADJ^68^. We used all three different approaches to test for robustness of our results. We present the results of the A1 approach in the main paper as these were considered robust and suitable for macroevolutionary analyses by Qian and Jin^63^. Using this approach, we randomly selected 20 phylogenetic trees for our analyses. The results based on phylogenetic trees built according to A2 and A3 are presented in Supplementary Fig. 11.

To test for robustness of our results, we ran all analyses with a second source of dated seed plant phylogenies from Smith and Brown^65^ (Supplementary Fig. 8). From their data sources, we used the two phylogenies (ALLOTB and ALLMB) that are based on available Genbank sequences and the Open Tree of Life synthetic tree^69^, with a backbone provided either by the Open Tree of Life version 9.1^70^ (ALLOTB) or by Magallon et al.^71^ (ALLMB). Detailed procedure of tree inference and differences between the two backbones are provided in the original publication^65^. We checked for concordance in species names between the AFE data and the tips of the phylogenetic trees. We removed all species in the phylogenies not included in the AFE data set and removed all AFE species not found in the phylogenetic trees. This resulted in a total of 3459 angiosperm and 39 gymnosperm species for the ALLOTB trees, and 3458 angiosperm and 39 gymnosperms for the ALLMB trees, respectively.

### Climate data and environmental distance analysis

For the current climate, we downloaded the 19 bioclim variables from Worldclim (version 1.4) at a resolution of 10 arcmin, cropped them to the study area, and stacked them into a raster stack using the raster R-package^72^. We then reprojected and spatially aggregated the stack to the projection of the distribution files (LAEA) at a spatial resolution of 50 km, the native resolution of the AFE distribution data. To calculate environmental distances among data points, we extracted the values from the stack for all AFE points, standardized the bioclimatic values, and ran a PCA (princomp function in R) with all 19 standardized bioclim variables. For further analyses, we used the first six PCA axes that cumulatively explained 98.0% of the variance among the 19 bioclim variables. Finally, environmental distances among AFE points were calculated as the Euclidean distance using the first six PCA axis values.

### Velocity of climate change (Vocc)

The climate change velocity indicates the rate at which climate is displacing spatially on a yearly basis. Traditionally, two forms of Vocc calculation have emerged, a distance-based and a gradient-based approach^73^. To calculate the velocity of climate change since the LGM (Vocc), we used the gradient-based approach using the gVocc function provided in the VoCC R-package^35,73^ and generated Vocc similarly as was done in a previous study^74^. This approach calculates the climate change velocity, gVocc (in km/year), as the ratio of the temporal climate trend, *s* (in °C/year for temperature or mm/year for precipitation), taken from the slope of a simple linear regression of the local (cell-wise) climatic time series, to the local spatial climatic gradient, *g* (in °C/year for temperature or mm/year for precipitation), based on a 3 × 3 cell neighborhood^74^ as gVoCC = *s*∕*g*. For our calculations, we used paleoclimate data for annual mean temperature and annual precipitation sum (with a resolution of 10 arcmin) which were available for Europe in 1000-year time steps back to the last glacial maximum (LGM; 21,000 years BP) based on monthly climate data, generated by Maiorano and colleagues^75^. This is the same dataset as was used in the climate stability and refugia analyses. We calculated the velocity of climate change from 1000 to 21,000 years BP independently for annual mean temperature and annual precipitation, and separately for 1000-years time steps. The Vocc layers of each climate variable were then averaged across the period from LGM to present and reprojected to the LAEA projection at a 50 km resolution. Next, we converted negative velocities (standing for local cooling or drying) into absolute velocities and calculated the maximum velocity per cell across both climate layers (to combine the velocities from the two climate layers into one single Vocc layer). While 99.5% of the data had realistic Vocc values since LGM of 0.5–1 km/year, we identified outliers with unrealistic values reaching 100 km/year. To remove such extreme outliers, we reset the values of these 16 pixels (0.5% of the 3116 analyzed land pixels) to the 99.5th percentile value in Vocc. We then log-transformed the Vocc values to enable better separation of very low to intermediate Vocc values. Finally, we applied a low-pass filter of climate change velocity in a 5 × 5 cell window because climate change velocity is strongly affected by local topographic slope angles, which are of smaller spatial extent than the range data from AFE. Naturally, this index is lower in mountainous terrain, because the distance to find the same climate after 1000 years is much closer compared to plains, where the spatial distance to find comparable climate in response to climate change is much larger. We used this Vocc layer as potential explanatory variable of found phylo-*β* patterns in Europe.

### Climate stability (ClimStab)

To measure climate stability, we applied the functions provided in the climateStability R-package^76^. We first measured the climate variability through time for annual mean temperature and annual precipitation sum using the same paleoclimate data with 1000-year time slices^75^ as were used in climate change velocity calculations (described above). Variability was calculated as the climate difference between time slices expressed as standard deviation, and then the mean among all time slices was computed. We then inverted variability to stability (as stability = 1/variability) separately for temperature and precipitation, rescaled both stability maps between 0 and 1, combined the temperature and precipitation stability maps into a rescaled climate stability estimate by taking the product of the two maps, and rescaled the final map again to values ranging from 0 to 1, all following^76^. The final climate stability map was reprojected to LAEA at a 50 km resolution. This map indicates average stability of climate jointly for temperature and precipitation between LGM and present at a temporal resolution of 1000 years.

### Refugia

We inferred for each species its potential refugial area using the KISSMig model^36^ by detecting cells, which: (1) were suitable at LGM based on a hindcasted species distribution model (see e.g., ref. ^67^), and (2) match with simulated migrations to reach species’ current distributions^36^. First, we calibrated logistic regression models (GLM) for each species by using its current distribution points (AFE points) as response (prevalence = 0.5 by weighting) and current annual mean temperature and annual mean precipitation as climate predictors (entering the model as linear and quadratic terms). The average performance of the initial GLMs evaluated by cross-validation and using the area under the ROC curve, AUC^77^, was 0.89 ± 0.083 for angiosperms and 0.88 ± 0.069 for gymnosperms, indicating that these comparably simple models successfully fitted current plant distributions and are thus useful for hindcasting their distribution to the LGM. For each species, we then projected its distribution under LGM climate and simulated migration from all suitable cells (i.e., suitablility > 0.5) separately for all 10 × 10 arcmin cells across Europe. To this end, we calculated suitability maps for 1000-year steps since LGM based on paleoclimate and the initial GLM. We then applied the KISSMig model^36^, which iteratively uses a simple 3 × 3 cell algorithm on top of these suitability maps to assess whether range expansion from each suitable LGM cells likely results in meaningful predictions of current species ranges. We ran KISSMig with a spatial resolution of 10 arcmin (c. 18.6 km) and used squared suitability values to fulfill basic empirical expectations (see https://www.archive.org/services/purl/wsl/kissmig). We retained all cells as potential refugial area if they were suitable at LGM and the presence–absence information of accessibility from these cells improved the performance (*D*^2^) of the initial GLM and the accessibility parameter had positive signs, meaning that accessibility from these cells better explained species presences when added as a predictor and this improvement was positively, not negatively, related to the current distribution^36^. Because potential migration rates are unknown for most species, we simulated one to ten KISSMig iterations per 1000 years, or 21–210 iterations since LGM corresponding to potential maximum migration rates of ca. 19 to 186 m/year. For final analysis, we used the refugial area of the migration rate for which the calculated accessibility explained current species occurrences best^36^. The average deviance (*D*^2^) explained increased from 0.48 to 0.68 for angiosperms, and from 0.44 to 0.67 for gymnosperms, respectively, after accounting for accessibility from refugial areas. The increase supports the adequacy of the model parametrization using annual temperature and precipitation for constraining the simulations of past migrations from candidate LGM refugial cells.

### Distance to refugia (DistRef)

For each species, we measured the minimum distance of each raster cell across Europe to its closest reconstructed refugial raster cell using the distance command in the raster R-package^72^ and calculated the mean (shortest) distance to refugia among all species present per sample location for all raster cells across Europe.

### Range size

We calculated the mean range size among all species occurring in a sample location by taking the mean of the number of occupied AFE points across Europe per species present in any given sample location.

### Phylo-*β* analyses

We calculated the true mean phylo-*β*^24,78^ from all pairwise dissimilarity calculations (Simpsons pairwise dissimilarity index) of a focal point to its 24 closest neighbors (see results for its 8 or 48 closest neighbors in Supplementary Fig. 12) using the betapart R-package^79^. To remove the effect of local geographic and environmental distance on phylo-*β*, we performed a generalized linear model with logit-transformed phylo-*β* as dependent variable and geographic and environmental distance (linear and quadratic) among sampled sites as explanatory variable. Geographic distance was removed because the AFE points are not exactly equally spaced. The residuals from these local regressions represent the phylo-*β* fraction that is independent of both geographic and environmental distance among neighboring sample locations (Supplementary Fig. 1). The residuals were interpolated to geographic space for visualization and further analyses. This remaining spatial phylo-*β* pattern (residuals) represents the larger scale variation across Europe that is independent of local topographic variation and is driven by other historical and contemporary drivers.

### Variable importance and correlation

We used standardized linear regression models to explain the residual phylo-*β* from (standardized) range size, distance to LGM refugia and velocity of climate change (linear and quadratic terms). Because distance to LGM refugia and climate stability were highly correlated (*R* = −0.77), we only used one of them in the model at a time and calculated a second model in which distance to refugia was replaced with climate stability. A full correlation matrix among the four predictors used is given in Supplementary Fig. 13. The model fit was reported as model *R*^2^. We calculated the variable importance using the lmg method implemented in the relaimpo R-package^80^ and rescaled the values to proportions summing up to 100% by adding up contributions from linear and quadratic terms per variable, and finally plotted them as barplot for assessing their relative contributions.

### Mapping of results across Europe

All spatial results were mapped at the same standard 50 km resolution (LAEA projection) and illustrated for better orientation over a hillshade of the European topography^81^ using semitransparent color scales.

### Reporting summary

Further information on experimental design is available in the Nature Research Reporting Summary linked to this paper.

## Supplementary information

Supplementary Information

Peer Review File

Reporting Summary

## Data Availability

The source data underlying Figs. 1–4 are provided as a Source Data file, which is available from the Dryad Digital Repository: 10.5061/dryad.j6q573n8t. The original backbone phylogeny (PhytoPhylo) is available from Qian and Jin^63^. The AFE distribution data is under publisher’s copyright and can be requested from *Atlas Florae Europaeae*^58,59^ (https://www.luomus.fi/en/database-atlas-florae-europaeae). The paleoclimate data for annual mean temperature and annual precipitation is online available at: https://www.archive.org/services/purl/wsl/kissmig.

## Code availability

All analyses were conducted in the R environment^62^ (version 3.5.1). The packages and codes used include Taxonstand^61^ (version 2.1), ape^82^ (version 5.3), stringr^83^ (version 1.3.1), S.PhyloMaker^63^, FNN^84^ (version 1.1.2.1), raster^72^ (version 2.9.23), rgdal^85^ (version 1.3.4), VoCC^73^ (version 1.0.0), climateStability^76^ (version 0.1.1), kissmig^36^ (version 1.0.3), betapart^79^ (version 1.5.1), relaimpo^80^ (version 2.2.3), RColorBrewer^86^ (version 1.1.2), and fields^87^ (version 9.6). Code generated for analyses and illustrations is available from the corresponding author.
