# Supplementary material for: Rapid climate change results in long-lasting spatial homogenization of phylogenetic diversity
Source: Nat Commun. 2020 Sep 16;11:4663. doi: 10.1038/s41467-020-18343-6 (PMC7495423; doi:10.1038/s41467-020-18343-6)
Supplement: Supplementary file 1 — Supplementary Information [file 41467_2020_18343_MOESM1_ESM.pdf]

Supplementary Information

# Rapid climate change results in long-lasting spatial homogenization of phylogenetic diversity

Saladin et al.

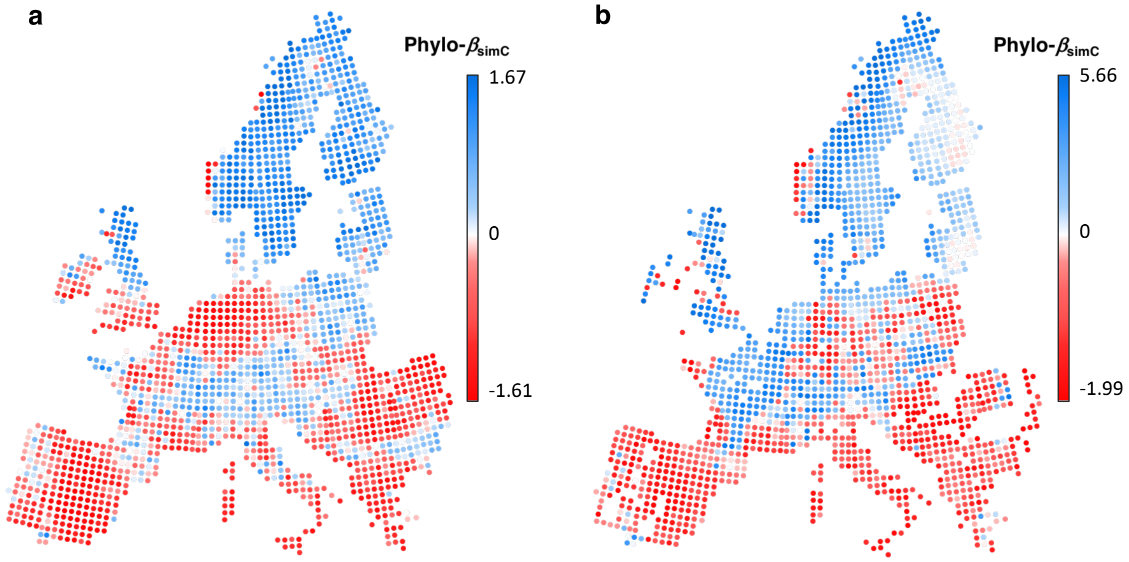


**Supplementary Fig. 1 l Phylogenetic turnover, phylo–$\beta_{simC}$, at original AFE sample locations. a-b**, patterns of phylo–$\beta_{simC}$ prior to interpolating them to a 50 km raster across Europe for angiosperms (**a**) and gymnosperms (**b**). The patterns match well with the interpolated surfaces in the main text. The sample locations represent the centroids of the quasi-rectangular AFE polygons for which we compiled the species list for phylogenetic turnover analyses.

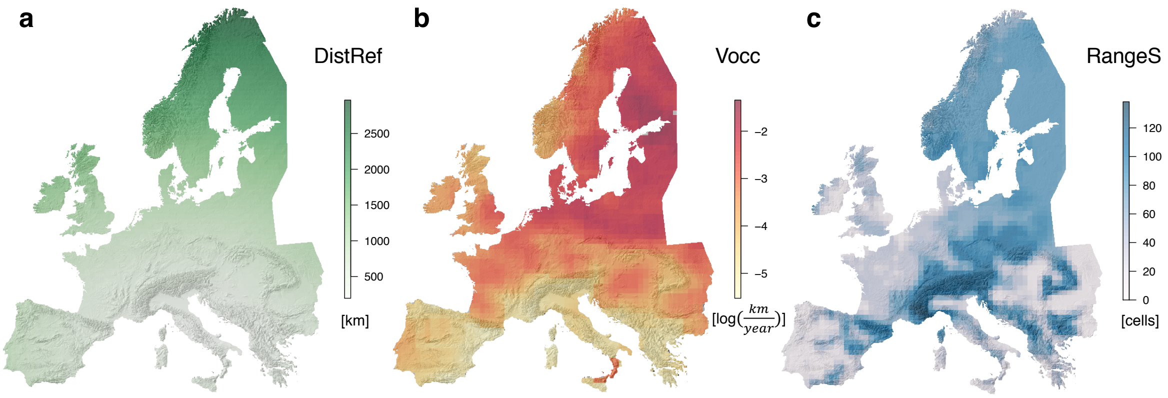


**Supplementary Fig. 2 | Explanatory variables of gymnosperm phylo-$\beta_{simC}$. a**, Mean distance to refugia (DistRef) of gymnosperms was calculated as the average distance among all species per cell to its reconstructed LGM distribution limits (see Fig. 4 for species' LGM distributions). **b**, The velocity of climate change (Vocc) since the LGM was calculated based on 1000-year time steps of reconstructed temperature and precipitation trends since the LGM. Vocc considers both the rate of climate change and the rate of elevation change of the local topography and thus expresses the average spatial change of climatic variables over time from the LGM to the present. **c**, Mean range size (RangeS) of the local species pool of gymnosperms that was calculated as the average range size of all species per cell across their European distribution as mapped in the Atlas Flora Europaea. This range size underestimates the true size of species expanding beyond Europe. The underlying hillshade maps of Europe are based on the digital elevation model of the European Environment Agency[1].

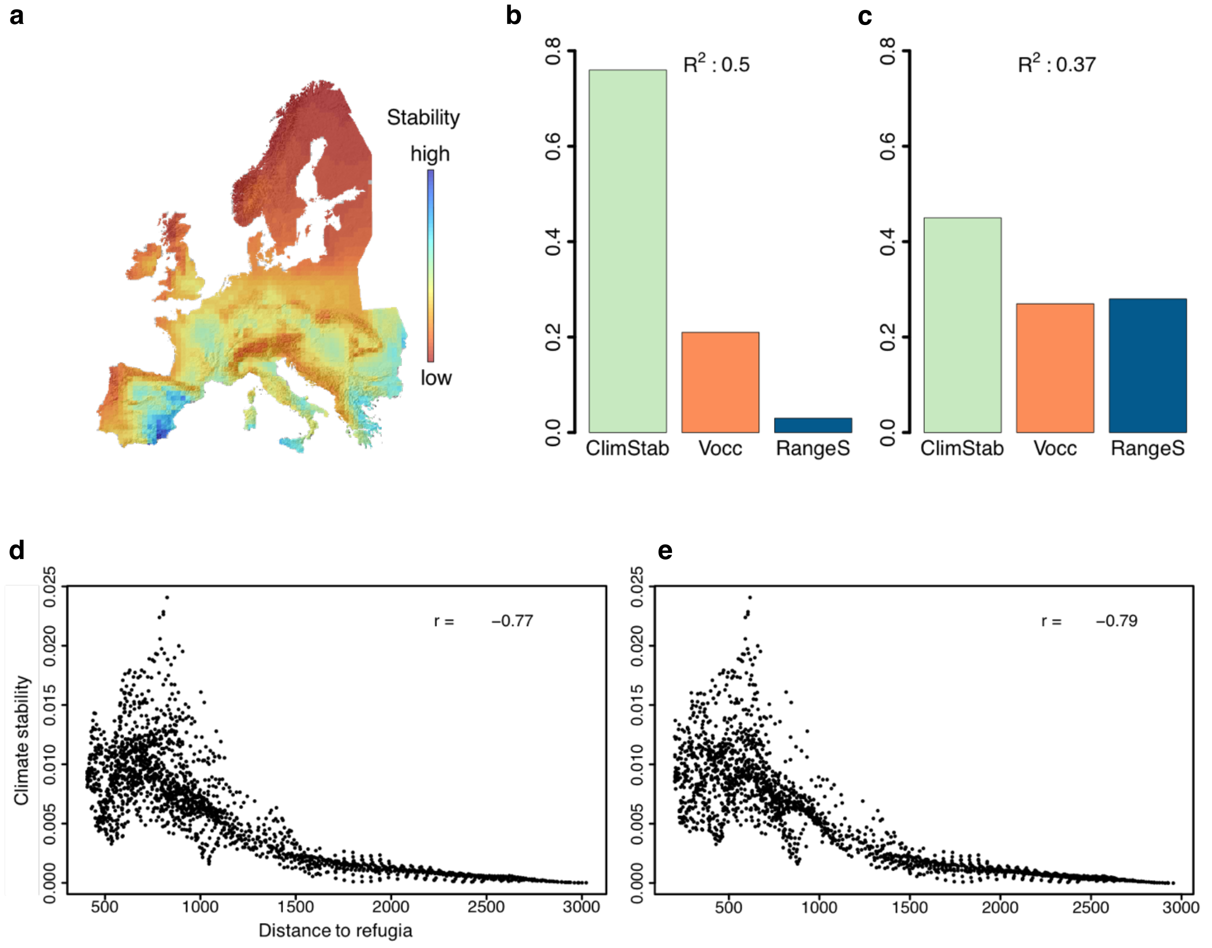


**Supplementary Fig. 3 | Climate stability (ClimStab) since the LGM and relative variable importance on phylo–$\beta_{simC}$ and variable correlation.** **a**, The stability of changing climates was calculated based on 1000-year time steps of reconstructed temperature and precipitation trends since the LGM. ClimStab only considers the rate of climate change and does not consider and the rate of elevation change of the local topography. It thus expresses the average temporal trend in temperature and precipitation over time since the LGM. The underlying hillshade map of Europe is based on the digital elevation model of the European Environment Agency[1]. **b**, Variable importance of climate stability since LGM (ClimStab), velocity of climate change (Vocc) and range size (RangeS) in explaining phylo–$\beta_{simC}$ for angiosperms (**b**) and gymnosperms (**c**). $R^2$ represents the model fit of a standardized linear regression to explain phylo–$\beta_{simC}$ by means of RangeS, ClimStab and Vocc (linear and quadratic terms). Relative variable importance was re-scaled to sum to 1.0. **d-e**, Correlation between distance to refugia (used in the main text) and climate stability with associated correlation coefficient for angiosperms (**d**) and gymnosperms (**e**). Only distance to refugia was used in the main text because DistRef and ClimStab are highly correlated.

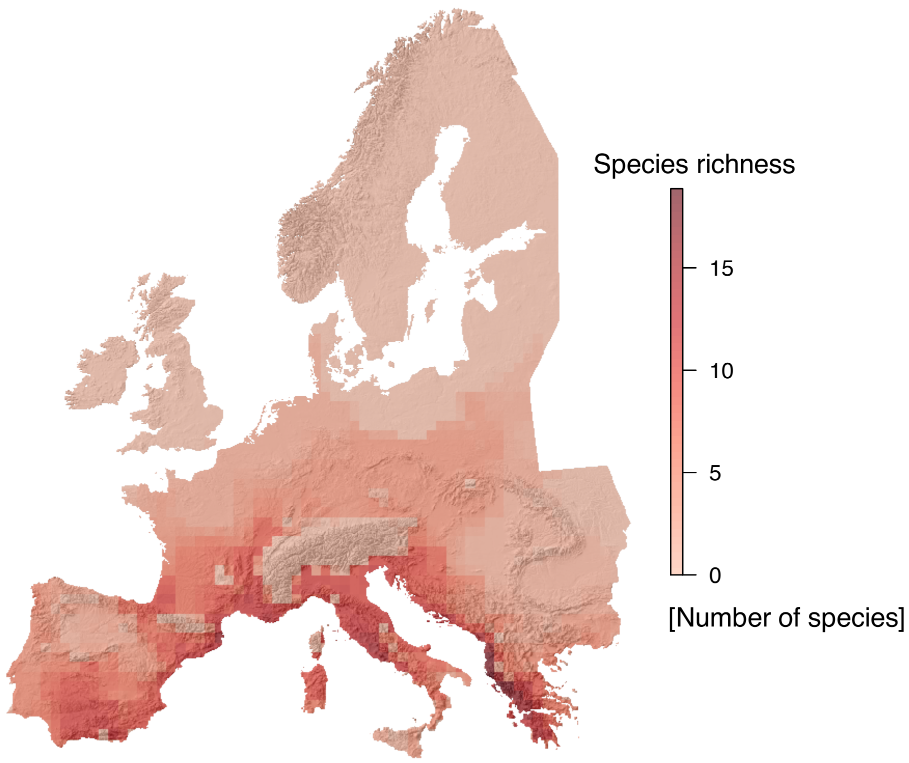


**Supplementary Fig. 4 I Gymnosperm species richness during the last glacial maximum (LGM).** Richness was inferred from dynamic KISSMig simulations for each species individually and represents the accumulated richness per 50 km cell. The underlying hillshade maps of Europe are based on the digital elevation model of the European Environment Agency[1].

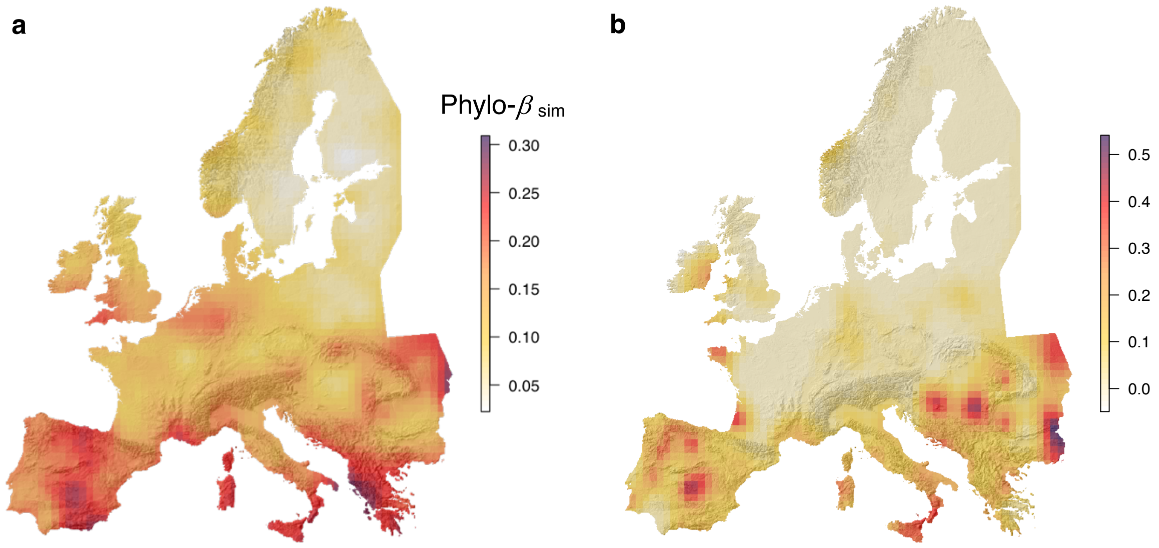


**Supplementary Fig. 5 | True phylogenetic turnover, phylo–$\beta_{sim}$. a-b**, The true phylogenetic turnover based on the Simpson formula (phylo–$\beta_{sim}$) and calculated among 24 neigboring cells relative to the focal cell represents the richness-corrected turnover, meaning that it is not affected by richness differences among neighboring cells. This phylo–$\beta_{sim}$ metric, plotted for angiosperms **(a)** and gymnosperms **(b)**, is further influenced by both environmental and geographic distances among neighboring cells (see Supplementary Figure 6) and larger scale historical effects independent of the regional neighborhood effects. The underlying hillshade maps of Europe are based on the digital elevation model of the European Environment Agency[1].

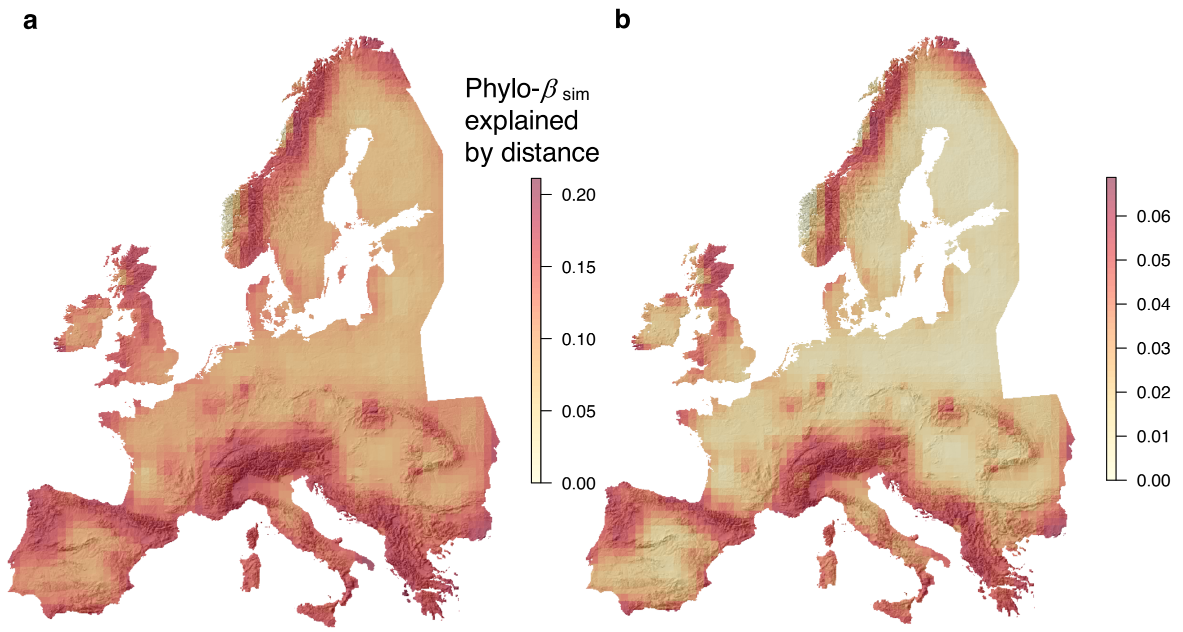


**Supplementary Fig. 6 | Size of removed distance effects. a-b**, Effect of geographic and environmental distances on phylo–$\beta_{sim}$ that is removed for calculation of distance-corrected phylo–$\beta_{simC}$ used in the main text (in units of phylo–$\beta_{sim}$). The effect metric, plotted for angiosperms **(a)** and gymnosperms **(b)**, represents the logistic regression model of explaining phylo–$\beta_{sim}$ by means of linear and quadratic terms for the mean geographic and environmental distance among neighboring cells relative to each focal cell. This effect is removed by taking the residuals from this model, represented as phylo–$\beta_{simC}$ in the main text. The underlying hillshade maps of Europe are based on the digital elevation model of the European Environment Agency[1].

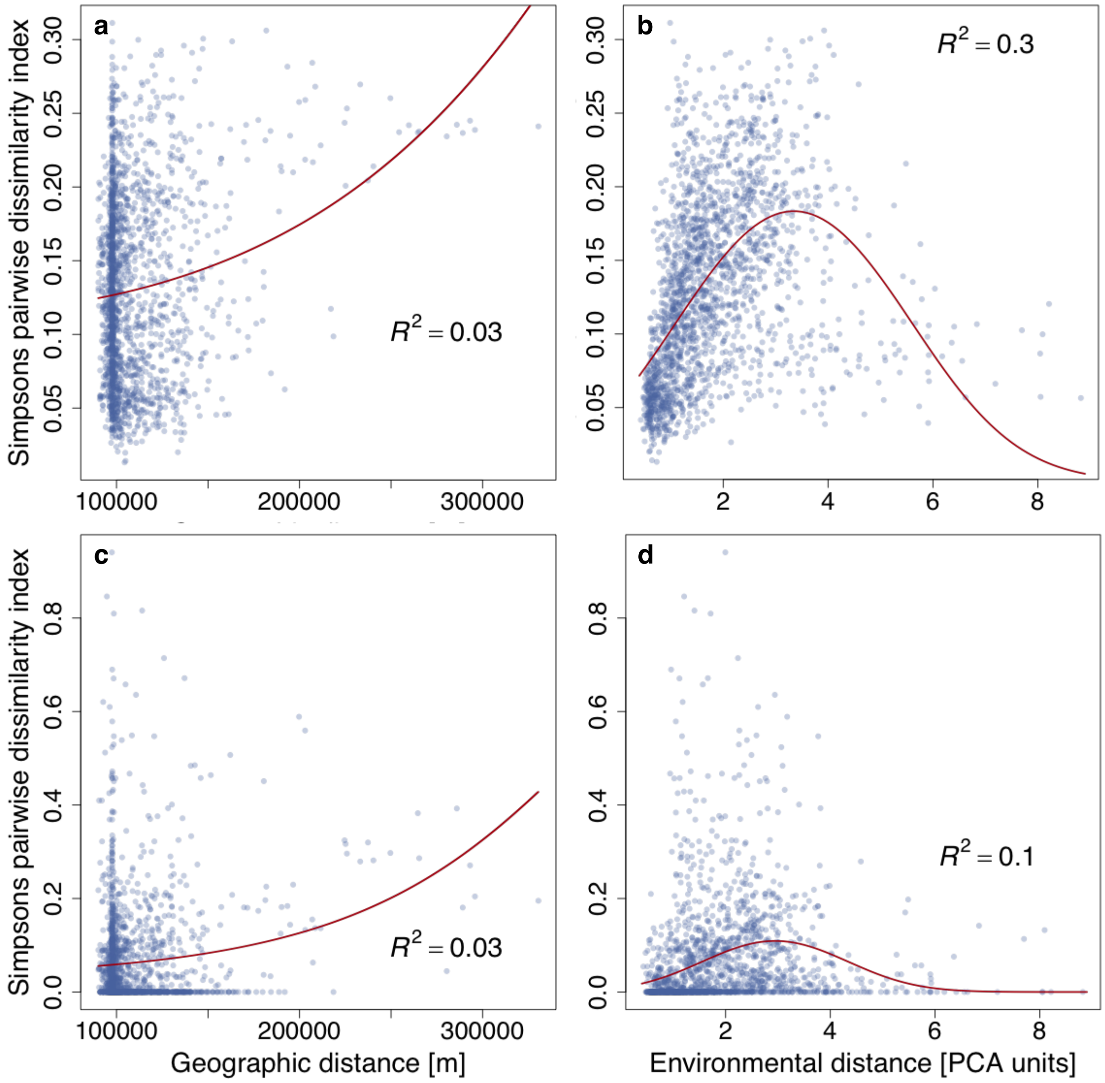


**Supplementary Fig. 7 | Regression of true phylogenetic turnover, phylo–$\beta_{sim}$, against geographic and environmental distances. a-d,** True phylogenetic turnover phylo–$\beta_{sim}$ among neighboring cells is regressed against geographic **(a,c)** and environmental **(b,d)** distance among cells for angiosperms **(a-b)** and gymnosperms **(c-d)**. This distance effect is removed by means of a generalized linear model using linear and quadratic terms and the residual turnover represents the regional, corrected phylogenetic turnover, phylo–$\beta_{simC}$, which is corrected for effects of geographic and environmental distances.

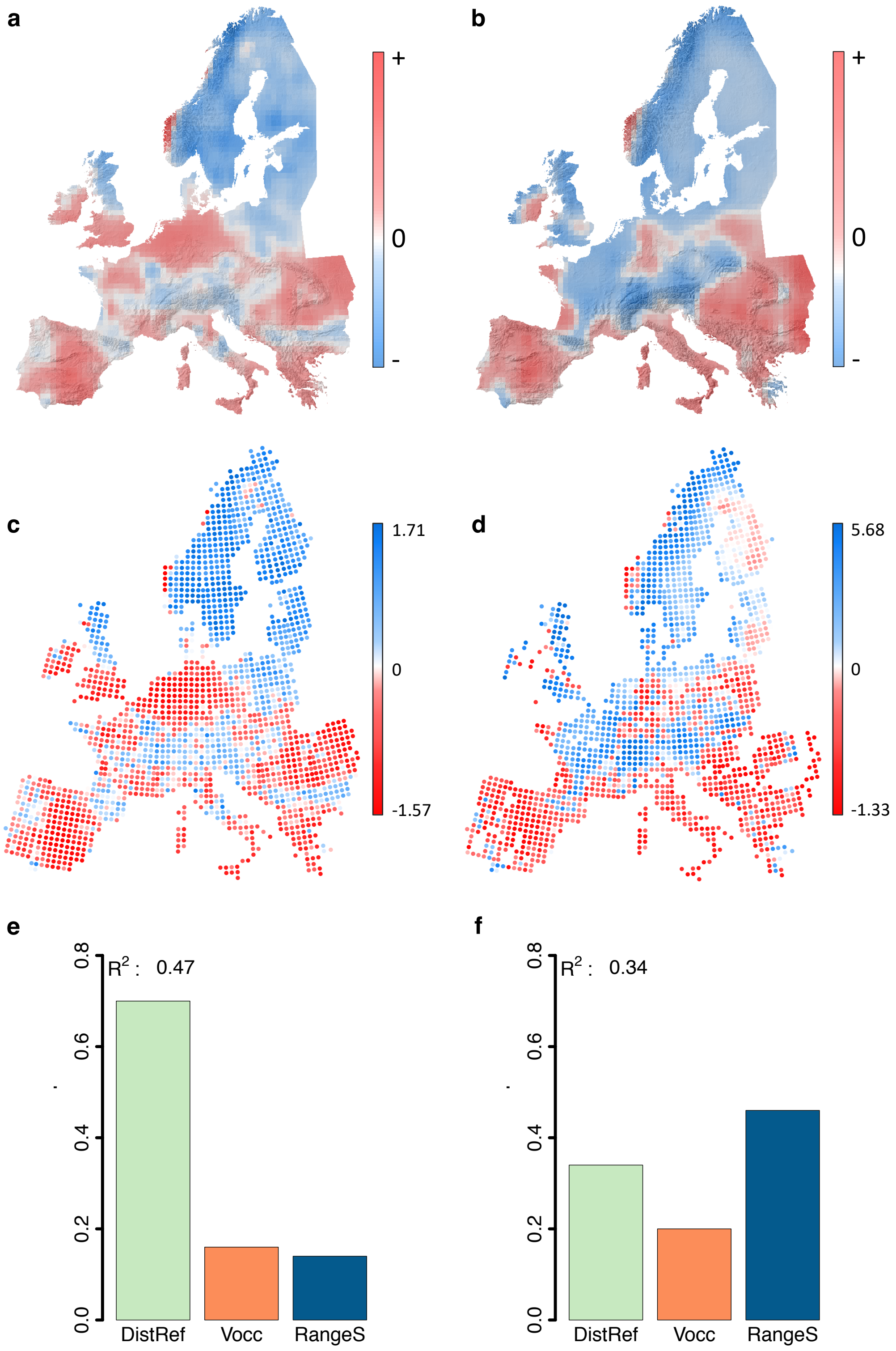
a
+
0
-
b
+
0
-
c
1.71
0
-1.57
d
5.68
0
-1.33
e
$R^2$: 0.47
0.0
0.2
0.4
0.6
0.8
DistRef
Vocc
RangeS
f
$R^2$: 0.34
0.0
0.2
0.4
0.6
0.8
DistRef
Vocc
RangeS

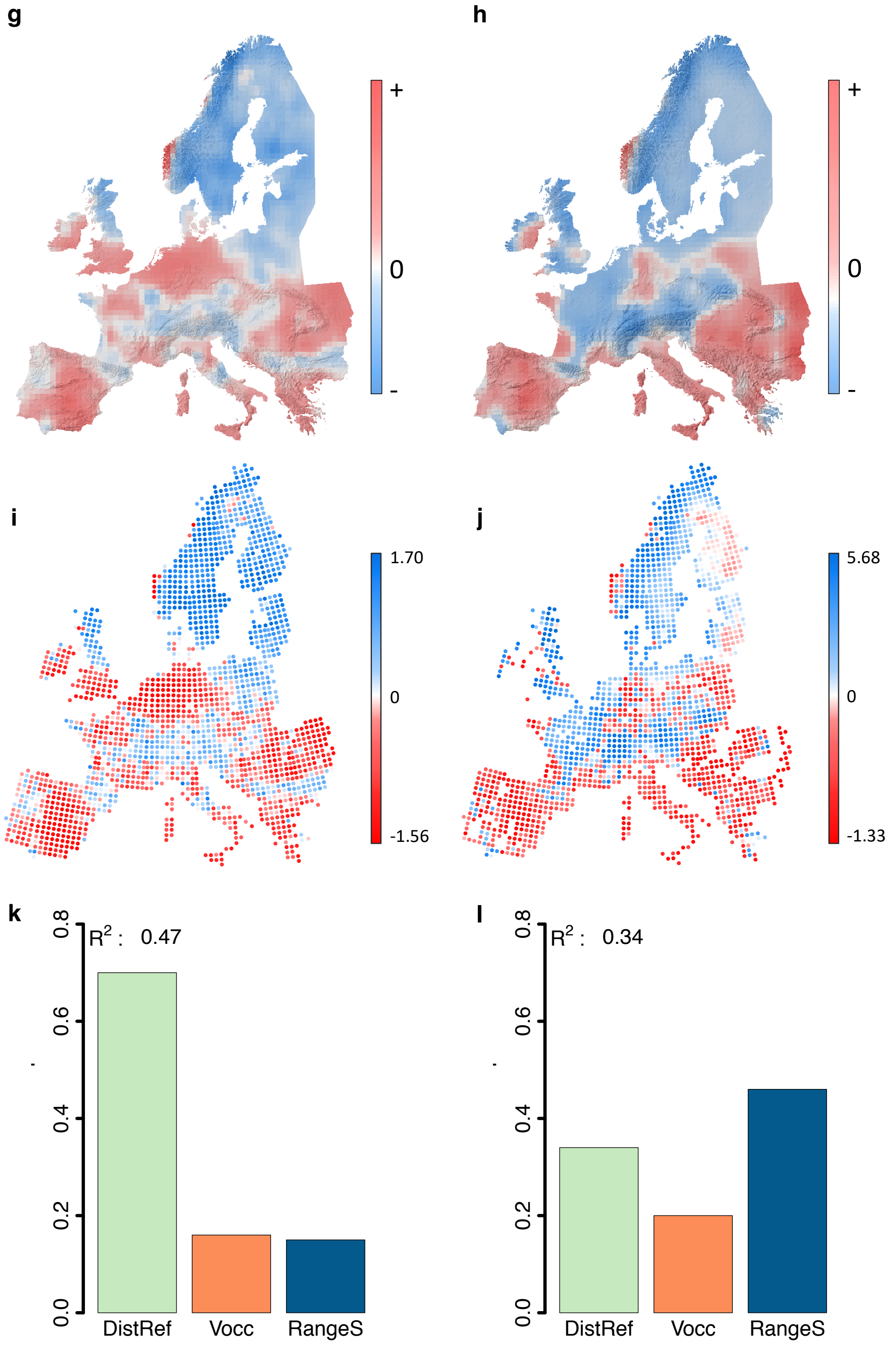


**Supplementary Fig. 8 | Robustness analysis of the main result based on alternative phylogenetic trees (Smith and Brown, 2018)[2].** Patterns of distance-corrected phylo–$\beta_{simC}$ for angiosperms (**a,c,g,i**) and gymnosperms (**b,d,h,j**) when using the *ALLMB*-phylogenetic tree (**a-d**), or the *ALLOTB*-phylogenetic tree (**g-j**). Variable importance of distance to refugia (DistRef), velocity of climate change (Vocc) and range size (RangeS) in explaining phylo–$\beta_{simC}$ for angiosperms (**e,k**) and gymnosperms (**f,l**) when using the *ALLMB*-tree (**e,f**) and using the *ALLOTB*-phylogenetic tree (**k,l**). The underlying hillshade maps of Europe are based on the digital elevation model of the European Environment Agency[1].

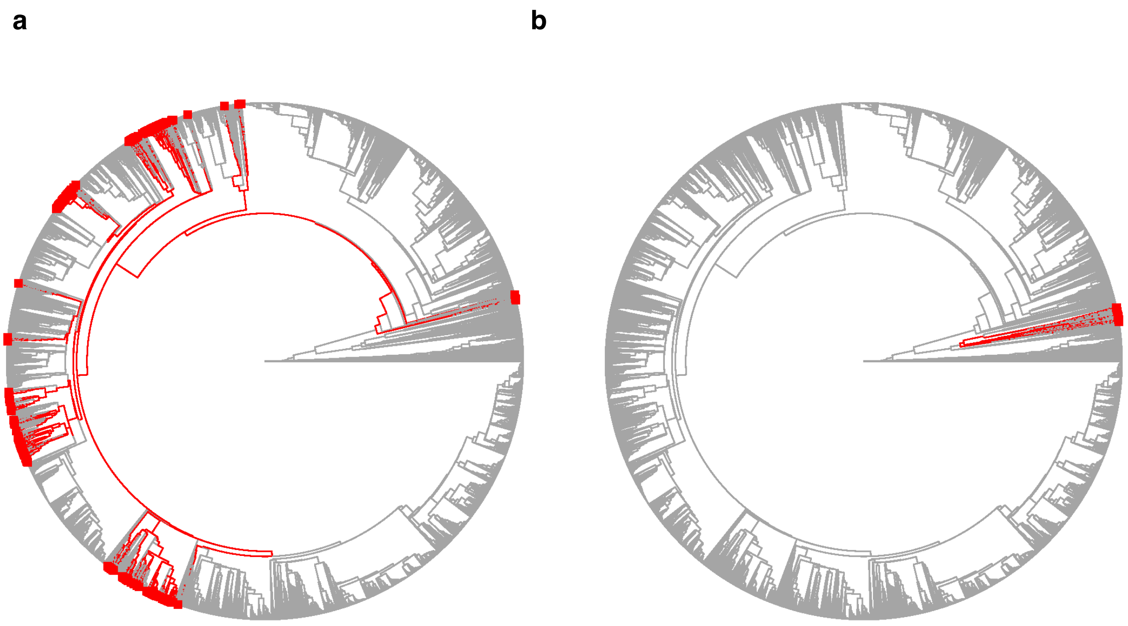


**Supplementary Fig. 9 l Phylogentic coverage of the taxa available in the AFE dataset.**
**a-b,** species available in the AFE dataset are marked in red for angiosperms **(a)** and gymnosperms **(b)** and plotted on the global, species-level plant megaphylogeny (PhytoPhylo) by Qian and Jin[3] plotted in gray color. Note that PhytoPhylo contains seed plant clades not available in Europe and also clades other than seed plants.

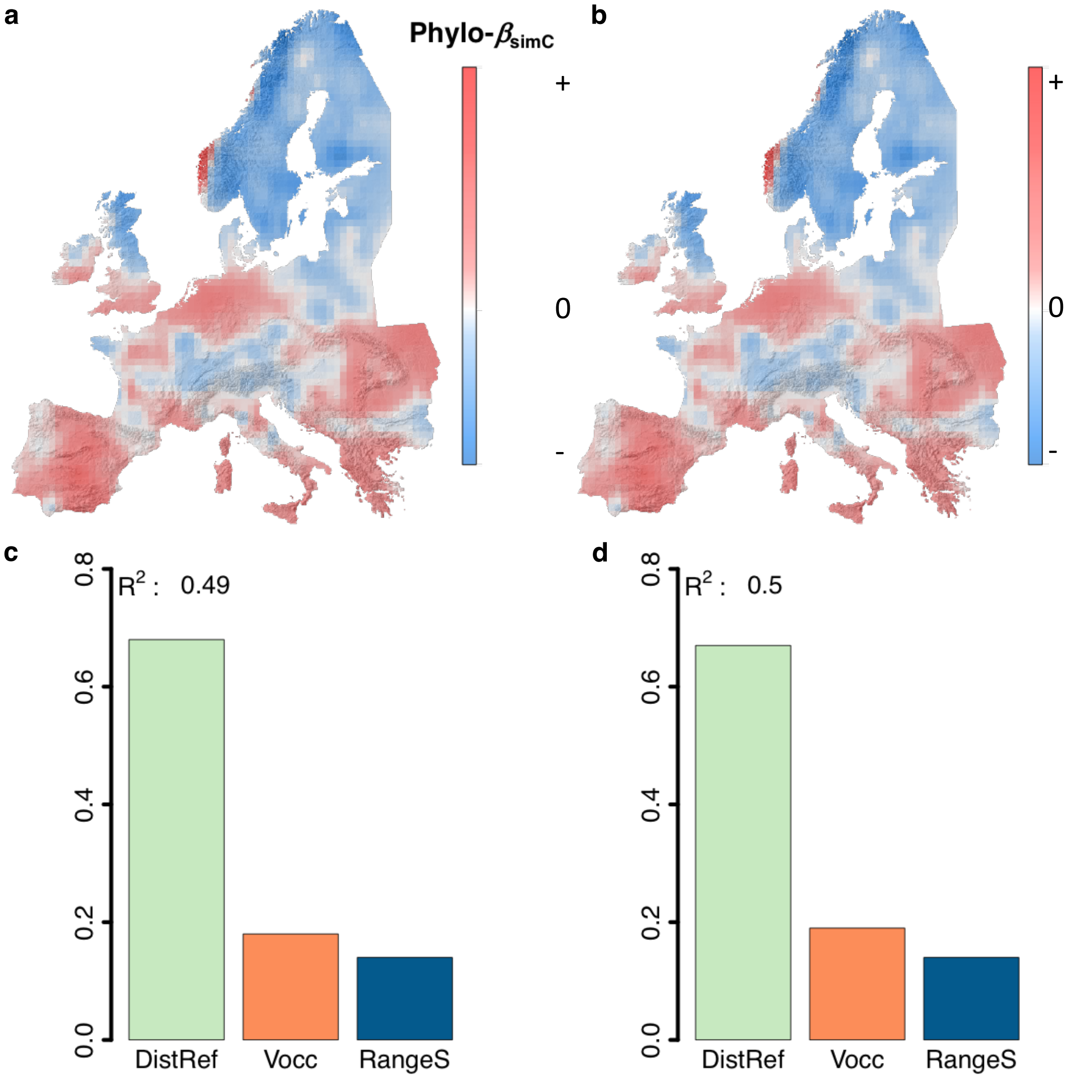


**Supplementary Fig. 10 l Robustness analysis of the angiosperm main result when adding removed species randomly to the respective families. a-b**, Patterns of distance-corrected phylo–$\beta_{simC}$ for angiosperms as used in the main text (**a**) and the extended set of angiosperms (**b**) where the species, whose phylogenetic position within families was ambiguous (see Supplementary Table S1), were added as polytomies to the respective family. **c-d**, Variable importance of distance to refugia (DistRef), velocity of climate change (Vocc) and range size (RangeS) in explaining phylo–$\beta_{simC}$ for angiosperms as used in the main text (**c**) and with the modified angiosperm set (**d**) using the phylogenetic trees with those species added that were not unambiguously placed in the phylogeny used in the main text. The underlying hillshade maps of Europe are based on the digital elevation model of the European Environment Agency[1].

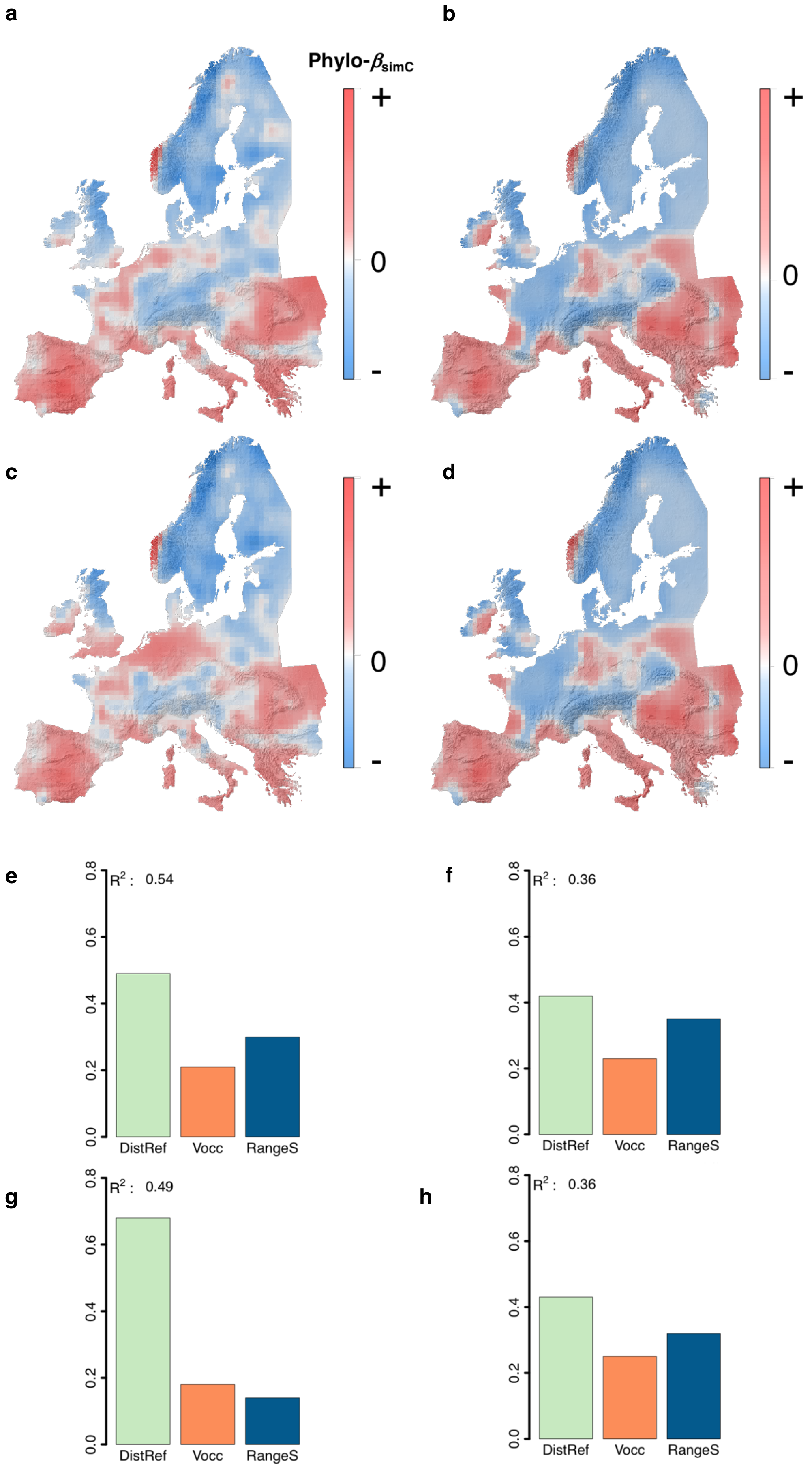
a
b
c
d
Phylo-βsimC
+
0
-
e
f
g
h
R2: 0.54
R2: 0.36
R2: 0.49
R2: 0.36
0.0
0.2
0.4
0.6
0.8
DistRef
Vocc
RangeS

**Supplementary Fig. 11 I Robustness analysis of the main result when using different methods to generate phylogenetic trees in PhyloMaker. a-d**, Patterns of distance-corrected phylo–$\beta_{simC}$ for angiosperms (**a,c**) and gymnosperms (**b,d**) when species, were added to the genus tree using the A2 (**a-b**) or the A3 (**c-d**) method in PhyloMaker (see method description in the main text). **e-h**, Variable importance of distance to refugia (DistRef), velocity of climate change (Vocc) and range size (RangeS) in explaining phylo–$\beta_{simC}$ for angiosperms (**e,g**) and gymnosperms (**f,h**) using trees where species were added to the genus using the S2 (**e-f**) or the S3 (**g-h**) method in PhyloMaker. The underlying hillshade maps of Europe are based on the digital elevation model of the European Environment Agency[1].

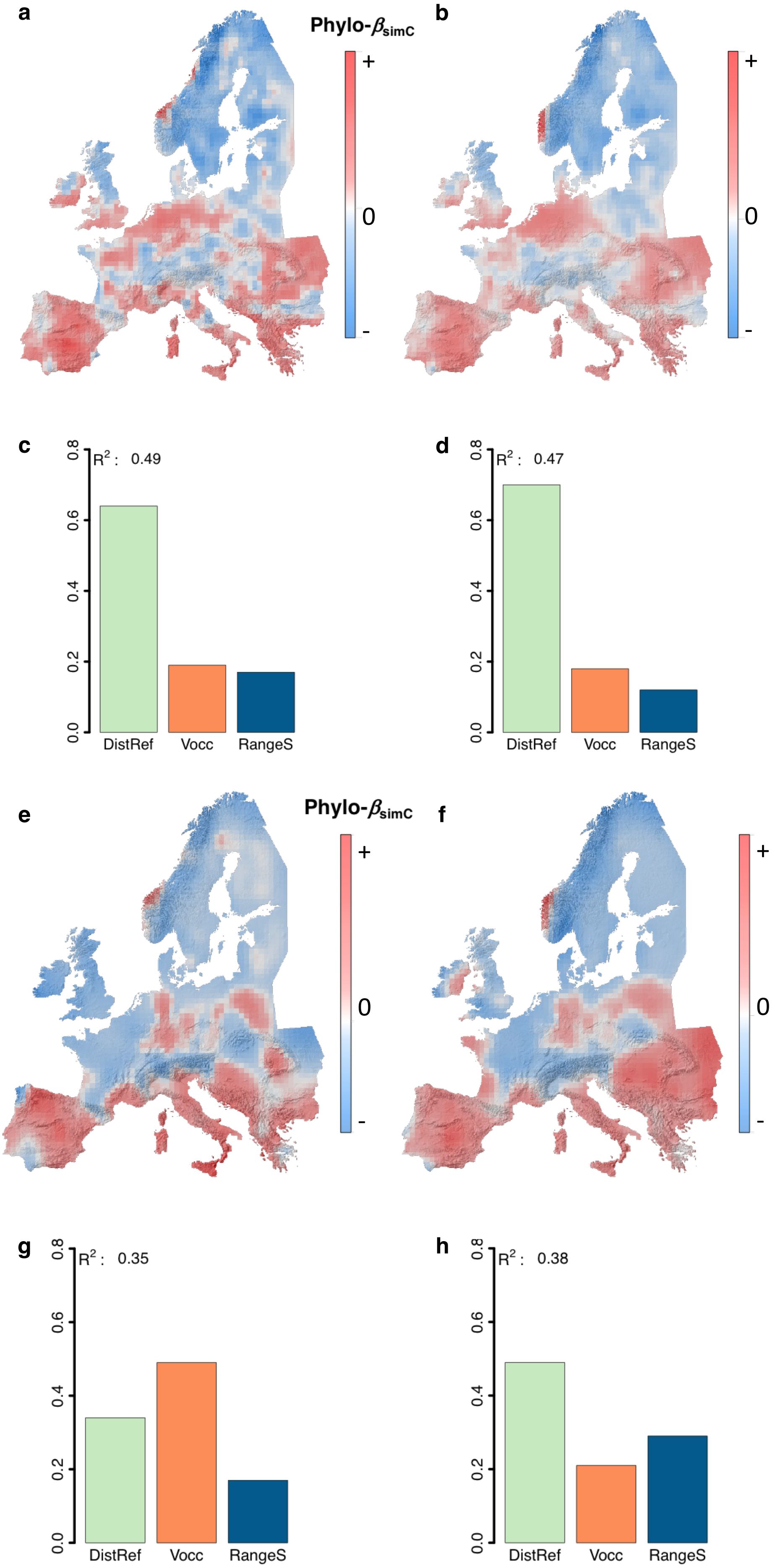

a
Phylo-$\beta_{simC}$
+
0
-
b
+
0
-
c
$R^2$: 0.49
0.0
0.2
0.4
0.6
0.8
DistRef
Vocc
RangeS
d
$R^2$: 0.47
0.0
0.2
0.4
0.6
0.8
DistRef
Vocc
RangeS
e
Phylo-$\beta_{simC}$
+
0
-
f
+
0
-
g
$R^2$: 0.35
0.0
0.2
0.4
0.6
0.8
DistRef
Vocc
RangeS
h
$R^2$: 0.38
0.0
0.2
0.4
0.6
0.8
DistRef
Vocc
RangeS

**Supplementary Fig. 12 | Robustness analysis of the main result when using different numbers of neighboring cells. a-h**, Patterns of distance-corrected phylo–$\beta_{simC}$ (a,b,e,f) and variable importance when explaining these phylo–$\beta_{simC}$ patterns (c,d,g,h) for angiosperms (**a-d**) and gymnosperms (**e-h**) when 8 (**a,c,e,g**) or 48 (**b,d,f,h**) neighbors are used to calculate mean phylo–$\beta_{simC}$ among neighboring cells. The results in the main text are calculated from 24 neighboring cells. The underlying hillshade maps of Europe are based on the digital elevation model of the European Environment Agency[1].

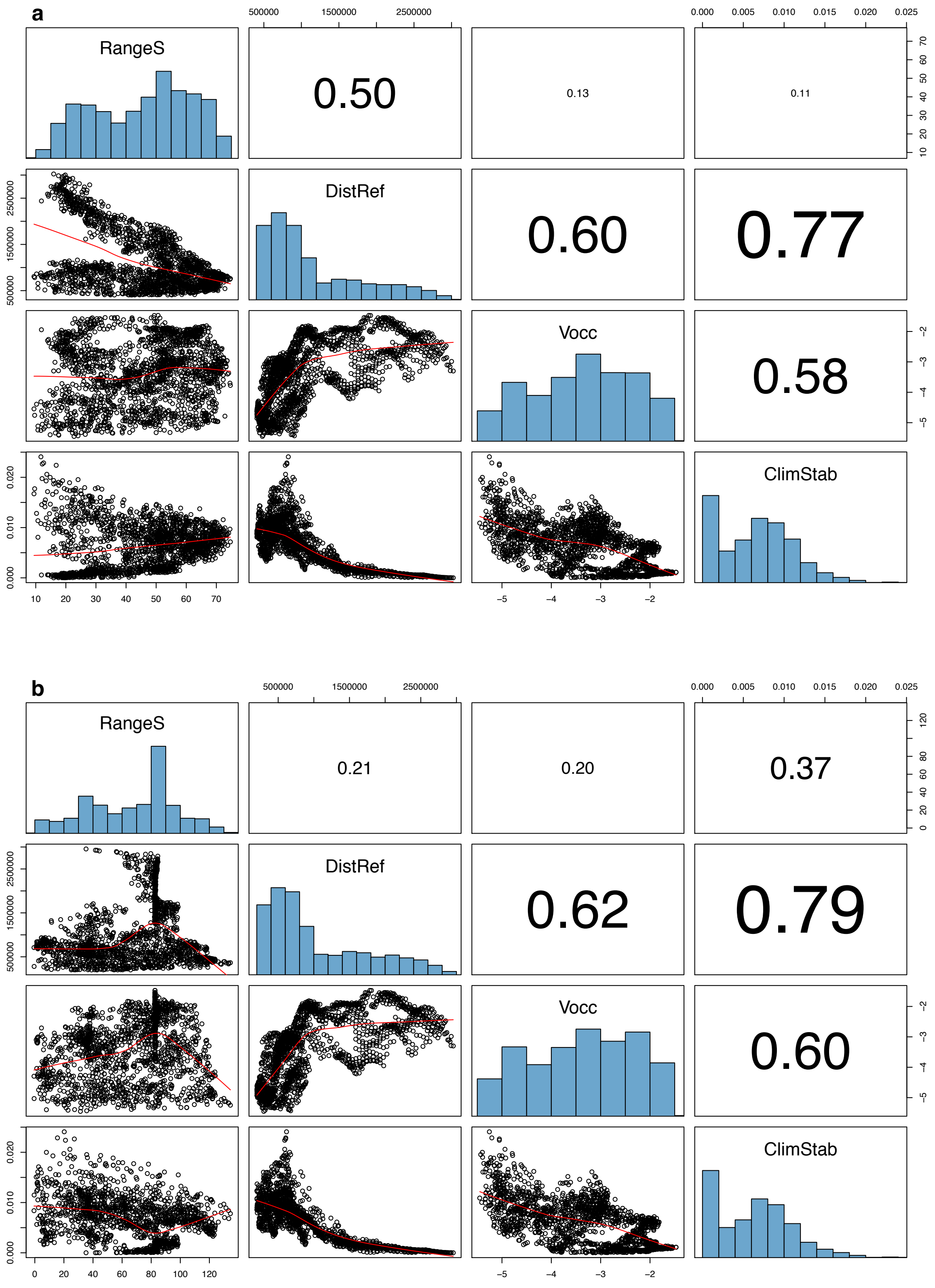


**Supplementary Fig. 13 | Correlation information among variables used to explain phylo–$\beta_{simC}$ patterns in Europe. a-b**, scattergram, distribution and Pearson correlation among four variables used to explain phylo–$\beta_{simC}$ across Europe sampled at angiosperm (a) and gymnosperm (b) sample points. Because few sample points of the Atlas Flora Europaeae (AFE) points contained now gymnosperms, and range size (RangeS) is species specific, the two plots differ among angiosperms and gymnosperms.

**Supplementary Table 1. I List of taxonomically ambiguous species.** This list represents species whose taxonomic position is ambiguous with respect to the used phylogenetic genus tree. These species were removed from analyses in the main text but added randomly to the respective family in the robustness analysis presented in Supplementary Figure 10.

| Family | Species | Family | Species |
|---|---|---|---|
| Amaranthaceae | Nanophyton erinaceum | Caryophyllaceae | Drypis spinosa |
| Amaranthaceae | Halimocnemis sclerosperma | Caryophyllaceae | Loeflingia baetica |
| Betulaceae | Corylus avellana | Caryophyllaceae | Loeflingia hispanica |
| Brassicaceae | Andrzeiowskia cardamine | Caryophyllaceae | Loeflingia tavaresiana |
| Brassicaceae | Biscutella morisiana | Caryophyllaceae | Ortegia hispanica |
| Brassicaceae | Bivonaea lutea | Caryophyllaceae | Chaetonychia cymosa |
| Brassicaceae | Boleum asperum | Caryophyllaceae | Bolanthus chelmicus |
| Brassicaceae | Bornmuellera dieckii | Caryophyllaceae | Bolanthus creutzburgii |
| Brassicaceae | Bornmuellera tymphaea | Caryophyllaceae | Bolanthus fruticulosus |
| Brassicaceae | Bornmuellera baldaccii | Caryophyllaceae | Bolanthus graecus |
| Brassicaceae | Degenia velebitica | Caryophyllaceae | Bolanthus intermedius |
| Brassicaceae | Erysimum degenianum | Caryophyllaceae | Bolanthus laconicus |
| Brassicaceae | Guiraoa arvensis | Caryophyllaceae | Bolanthus thessalus |
| Brassicaceae | Kernera saxatilis | Caryophyllaceae | Bolanthus thymifolius |
| Brassicaceae | Maresia nana | Crassulaceae | Rosularia serrata |
| Brassicaceae | Morisia monanthos | Crassulaceae | Pistorinia breviflora |
| Brassicaceae | Peltaria alliacea | Crassulaceae | Pistorinia hispanica |
| Brassicaceae | Peltaria emarginata | Cytinaceae | Cytinus ruber |
| Brassicaceae | Rhizobotrya alpina | Cytinaceae | Cytinus hypocistis |
| Brassicaceae | Succowia balearica | Molluginaceae | Corrigiola litoralis |
| Brassicaceae | Syrenia cana | Papaveraceae | Platycapnos saxicola |
| Brassicaceae | Syrenia montana | Papaveraceae | Platycapnos spicatus |
| Brassicaceae | Vella bourgaeana | Papaveraceae | Platycapnos tenuiloba |
| Brassicaceae | Vella lucentina | Papaveraceae | Platycapnos tenuilobus |
| Brassicaceae | Vella pseudocytisus | Papaveraceae | Pseudofumaria alba |
| Brassicaceae | Vella spinosa | Papaveraceae | Pseudofumaria lutea |
| Brassicaceae | Neurotropis platycarpa | Papaveraceae | Roemeria hybrida |
| Brassicaceae | Teesdaliopsis conferta | Papaveraceae | Rupicapnos africana |
| Brassicaceae | Sobolewskia sibirica | Papaveraceae | Sarcocapnos baetica |
| Brassicaceae | Sisymbrella aspera | Papaveraceae | Sarcocapnos crassifolia |
| Brassicaceae | Sisymbrella dentata | Papaveraceae | Sarcocapnos enneaphylla |
| Brassicaceae | Murbeckiella boryi | Papaveraceae | Sarcocapnos pulcherrima |
| Brassicaceae | Murbeckiella pinnatifida | Papaveraceae | Sarcocapnos saetabensis |
| Brassicaceae | Murbeckiella sousae | Papaveraceae | Ceratocapnos claviculata |
| Brassicaceae | Murbeckiella zanonii | Papaveraceae | Ceratocapnos heterocarpa |
| Brassicaceae | Lycocarpus fugax | Ranunculaceae | Callianthemum anemonoides |
| Brassicaceae | Litwinowia tenuissima | Ranunculaceae | Callianthemum coriandrifolium |
| Brassicaceae | Ionopsidium acaule | Ranunculaceae | Callianthemum kernerianum |
| Brassicaceae | Ionopsidium albiflorum | Ranunculaceae | Ceratocephalus falcatus |
| Brassicaceae | Ionopsidium savianum | Ranunculaceae | Ceratocephalus testiculatus |
| Brassicaceae | Hugueninia tanacetifolia | Resedaceae | Sesamoides interrupta |
| Brassicaceae | Eremoblastus caspicus | Resedaceae | Sesamoides minor |
| Brassicaceae | Didesmus aegyptius | Resedaceae | Sesamoides prostrata |
| Brassicaceae | Clausia aprica | Resedaceae | Sesamoides purpurascens |
| Brassicaceae | Chrysochamela draboides | Resedaceae | Sesamoides spathulifolia |
| Caryophyllaceae | Bufonia macropetala | Resedaceae | Sesamoides suffruticosa |
| Caryophyllaceae | Bufonia paniculata | Rosaceae | Aremonia agrimonoides |
| Caryophyllaceae | Bufonia perennis | Rosaceae | Sibiraea laevigata |
| Caryophyllaceae | Bufonia stricta | Urticaceae | Soleirolia soleirolii |
| Caryophyllaceae | Bufonia tenuifolia | | |

## Supplementary References


1 EEA. *Elevation map of Europe* (2004); https://www.eea.europa.eu/data-and-maps/data/digital-elevation-model-of-europe.

2 Smith, S. A. & Brown, J. W. Constructing a broadly inclusive seed plant phylogeny. *Am J Bot* **105**, 302-314, (2018).

3 Qian, H. and Jin, Y. An updated megaphylogeny of plants, a tool for generating plant phylogenies and an analysis of phylogenetic community structure. *J Plant Ecol* **9**, 233-239, (2016).
