# Supplementary material for: Rapid climate change results in long-lasting spatial homogenization of phylogenetic diversity
Source: Nat Commun. 2020 Sep 16;11:4663. doi: 10.1038/s41467-020-18343-6 (PMC7495423; doi:10.1038/s41467-020-18343-6)
Supplement: Supplementary file 3 — Reporting Summary [file 41467_2020_18343_MOESM3_ESM.pdf]

nature research

Corresponding author(s): Bianca Saladin

Last updated by author(s): 2020/08/12

Reporting Summary

Nature Research wishes to improve the reproducibility of the work that we publish. This form provides structure for consistency and transparency in reporting. For further information on Nature Research policies, see Authors & Referees and the Editorial Policy Checklist.

Statistics

For all statistical analyses, confirm that the following items are present in the figure legend, table legend, main text, or Methods section.

n/a	Confirmed	
☐	☒	The exact sample size (*n*) for each experimental group/condition, given as a discrete number and unit of measurement
☐	☒	A statement on whether measurements were taken from distinct samples or whether the same sample was measured repeatedly
☒	☐	The statistical test(s) used AND whether they are one- or two-sided<br>*Only common tests should be described solely by name; describe more complex techniques in the Methods section.*
☐	☒	A description of all covariates tested
☐	☒	A description of any assumptions or corrections, such as tests of normality and adjustment for multiple comparisons
☒	☐	A full description of the statistical parameters including central tendency (e.g. means) or other basic estimates (e.g. regression coefficient) AND variation (e.g. standard deviation) or associated estimates of uncertainty (e.g. confidence intervals)
☒	☐	For null hypothesis testing, the test statistic (e.g. *F*, *t*, *r*) with confidence intervals, effect sizes, degrees of freedom and *P* value noted<br>*Give P values as exact values whenever suitable.*
☒	☐	For Bayesian analysis, information on the choice of priors and Markov chain Monte Carlo settings
☒	☐	For hierarchical and complex designs, identification of the appropriate level for tests and full reporting of outcomes
☐	☒	Estimates of effect sizes (e.g. Cohen's *d*, Pearson's *r*), indicating how they were calculated

Our web collection on statistics for biologists contains articles on many of the points above.

Software and code

Policy information about availability of computer code

Data collection	Data was obtained from different sources described in the "Data availability" section and the specific sections in the Method. Data processing was done in the open source R statistical environment (version 3.5.1).
Data analysis	All analyses were conducted in the R environment (version 3.5.1). The packages used are listed in the "Code availability" statement in the manuscript. R code generated for the analyses is available from the corresponding author upon request and without restrictions.

For manuscripts utilizing custom algorithms or software that are central to the research but not yet described in published literature, software must be made available to editors/reviewers. We strongly encourage code deposition in a community repository (e.g. GitHub). See the Nature Research guidelines for submitting code & software for further information.

Data

Policy information about availability of data

All manuscripts must include a data availability statement. This statement should provide the following information, where applicable:

- Accession codes, unique identifiers, or web links for publicly available datasets
- A list of figures that have associated raw data
- A description of any restrictions on data availability

The source data underlying Figs 1-4 are provided as a Source Data file, which is available from the Dryad Digital Repository: https://doi.org/10.5061/dryad.j6q573n8t (doi:10.5061/dryad.j6q573n8t). The original backbone phylogeny (PhytoPhylo) is available from Qian and Jin (Ref 59). The AFE distribution data is under publisher's copyright and can be requested from Atlas Florae Europaeae (https://www.luomus.fi/en/database-atlas-florae-europaeae). The paleoclimate data for annual mean temperature and annual precipitation is online available at: https://www.wsl.ch/en/services-and-products/software-websites-and-apps/kissmig.html#tabelement1-tab3.

Field-specific reporting

Please select the one below that is the best fit for your research. If you are not sure, read the appropriate sections before making your selection.

☐ Life sciences ☐ Behavioural & social sciences ☒ Ecological, evolutionary & environmental sciences

For a reference copy of the document with all sections, see nature.com/documents/nr-reporting-summary-flat.pdf

Ecological, evolutionary & environmental sciences study design

All studies must disclose on these points even when the disclosure is negative.

Study description	We calculated the phylogenetic turnover of European vascular plants in a 50 km geographic grid space (using a moving window approach) after removing environmental distance effects among spatially neighboring cells. This allowed us to assess the spatial structure of the remaining phylogenetic turnover variation which we interpreted based on indicators representing legacies of past and rapid climate changes.
Research sample	Our raw data consisted of 1970 sample points representing centroids of polygons of the distribution atlas of the "Atlas Florae Europaeae" (https://www.luomus.fi/en/database-atlas-florae-europaeae). For each polygon, the presence and absence of each species (see below) is recorded. We extracted the full list of present species for each sample location as our raw data.
Sampling strategy	The selection of 1970 sample cells is a subset of all available data points of the AFE distribution atlas. For this subset, we excluded points in Eastern Europe (Belarus, Russia, Ukraine, Turkey) because of lower data quality, and less connected islands (Iceland, Balearics and Malta). This set therefore includes all available data points of the AFE distribution atlas that are neither of lower sampling quality, nor disconnected islands (that may distort the turnover metrics).
Data collection	We extracted all data from existing literature and online databases that are open access. No additional data was collected in the context of this study.
Timing and spatial scale	The climate data covers 1000-year time steps back to the Last Glacial Maximum, ranging from current to 21'000 years BP. The spatial scale is Europe.
Data exclusions	We matched the species list of the distribution database with phylogenetic tip data for name consistency, and we excluded species that did not match. This was done prior to any further analyses and as a result, we retained 3904 (from originally 4003) angiosperm species and all 41 gymnosperm species.
Reproducibility	No experiments were conducted for this study and the Methods include careful descriptions of data considered, processing and analyses conducted. Generally, all analyses were performed within the R statistical environment,. The code will be made available from the corresponding author upon request. The data source for the figures is already available from Dryad (link can be retrieved from the "Data availability" section).
Randomization	This is not relevant to our study as we applied a moving window approach, sampling systematically the whole geographic space.
Blinding	does not apply.

Did the study involve field work? ☐ Yes ☒ No

Reporting for specific materials, systems and methods

We require information from authors about some types of materials, experimental systems and methods used in many studies. Here, indicate whether each material, system or method listed is relevant to your study. If you are not sure if a list item applies to your research, read the appropriate section before selecting a response.

Materials & experimental systems

n/a	Involved in the study
☒	☐ Antibodies
☒	☐ Eukaryotic cell lines
☒	☐ Palaeontology
☒	☐ Animals and other organisms
☒	☐ Human research participants
☒	☐ Clinical data

Methods

n/a	Involved in the study
☒	☐ ChIP-seq
☒	☐ Flow cytometry
☒	☐ MRI-based neuroimaging
