# Supplementary material for: Rapid climate change results in long-lasting spatial homogenization of phylogenetic diversity
Source: Nat Commun. 2020 Sep 16;11:4663. doi: 10.1038/s41467-020-18343-6 (PMC7495423; doi:10.1038/s41467-020-18343-6)
Supplement: Supplementary file 2 — Peer Review File [file 41467_2020_18343_MOESM2_ESM.pdf]

Editorial Note: This manuscript has been previously reviewed at another journal that is not operating a transparent peer review scheme. This document only contains reviewer comments and rebuttal letters for versions considered at *Nature Communications*.

Reviewers' comments:

Reviewer #1 (Remarks to the Author):

The paper deals with a very important topic and approaches this with an exciting approach using spatial phylogenetics and using a dated tree estimating plant movement and refugial areas. I am supportive of the work. But have many questions that are a result of some aspects of the manuscript that are poorly presented or unclear. Is this work on seed plants or all vascular plants? That is a big issue that needs to be clarified. I am also unclear on the phylogeny used and why - again was it all vascular plants or seed plants? The authors refer to both but most of the data seem to be angiosperms. The authors also need to clarify issues of the scale used (is this fine scale enough?) and the numbers of the specimens analyzed for each species (were enough locations used per species?). The work addresses a significant question and is highly relevant to present day as well as potential future plant distributions.

23-24 we evaluate…phylogeneti diversity of European vascular plants…. Vascular plants are noted-- But only angiosperms and gymnosperms are noted in the text

24. The effect past climate change insert of The effect of past climate change

85. We analyze phylogenetic diversity separately for angiosperms and gymnosperms. What about other vascular plants (see above)?

89-90 both angiosperms and gymnosperms... Again what about other vascular plants?

150-151. Clear spatial structure in phylogenetic turnover of vascular plants ... But only angiosperms and gymnosperms are noted. What about ferns and lycophytes?

161. Different mechanisms can explain...
Dispersibility ability is not ever discussed which seems surprising

179. Recolonization dynamics—. See comment above

195 projected future climate change is of equal if not larger intensity— refs?

200 the effect....have. Change to has

208. Distribution data of angiosperms and gymnosperms... What about the other vascular plants? See comments above

220 Isoetes. These are lycophytes (not seed plants)— which again begs the question—what about these and other non-seed vascular plants?

226. Names. Again what about non seed vascular plants?

238. Use of the Qian and Jin. See comment below

237-238. I think the most recent complete and updated tree is Smith and Brown 2018. The authors

should note why Smith and Brown was not used? Also, it is a seed plant phylogeny as is Zanne et al. - I am not sure how non seed plants (the other vascular plants) were added here? This is a consistent problem in this ms.

238. Qian and Jin is an update of Zanne et al — Zanne et al is a seed plant phylogeny and not for all vascular plants. How were other vascular plants added?

241 Phytophylo includes all families of extant seed plants. What was done for non seed plants?

249. Total number of angiosperm species is given. We need totals for all vascular plants used in the study and how that compares to the actual total number for Europe

268 resolution of 50 km. Other spatial phylogenetic studies are more fine grained than this. Why such a large scale?

methods—how many specimens were examined per species in these inferences? Total number of specimens, etc?

Reviewer #2 (Remarks to the Author):

I thoroughly enjoyed reviewing this paper, as it sheds light on an important topic in climate change research. Saladin et al. analyzed the effects of rapid climatic change during the Pleistocene on the phylogenetic diversity of vascular plants in Europe. The authors find that phylogenetic turnover is generally higher in Southern Europe (a region with low climate velocity) than Norther Europe, (a region strongly influenced by high climate velocity). The authors conclude the decrease in phylogenetic diversity across Northern Europe leads homogenization throughout the landscape due to unstable climates and distance from refugia. This manuscript is concise, well written, and provides an important contribution to the field. However, clarification on several key methodological points is needed, most notably the AFE database. Without this clarification of the analyses the paper is lacking. The points below could be considered to increase the impact of the paper.

General comments:

Line 174 “Refugial areas can be expected in, or near, regions of high phylo-bsimC”

This is interesting wording since I would expect regions of high phylo-bsimC to be located mostly in areas of refugia. e.g., which is the driver of the relationship? Here, it’s worded such that phylo-bsimC drives refugia, but in the introduction (lines 80-82), it’s worded such that refugial dynamics drive phylo-bsimC.

Lines 186-192: High turnover in Northern and central locations

Do the authors have any insight in why these areas have high phylogenetic turnover? They mention these areas correspond to previous research, but is there anything they can add about these areas specifically regarding mechanisms that could produce the observed patterns? They do this nicely with low turnover in high climate velocity regions.

It would be interesting to see the stacked refugia maps for angiosperms and gymnosperms generated in this study and how they relate to the already known sites (R1, R2, and R3).

Line 271: change sixth to six.

Methodological comments:

Lines 209-211: “We included plant records within the extent of the study area, totaling to 1970 sample points representing centroids of AFE polygons.”

Initial comment: I am quite confused about this - does this mean every species was represented by just one point at the center of their AFE polygon? If so, there seems to be a substantial mismatch between the number of species with distribution data and the number of species in the phylogeny. More detailed information is needed for the AFE polygons and what information they contain because I am not sure what is encompassed within this database.

Follow-up comment: Just before submitting the review, I noticed that the authors included a statement in their Nature Research “Reporting Summary” that “the presence and absence of each species is recorded for each of these sample points”. Nothing like this statement was included in the main paper, and it would have been useful to head off a lot of confusion about the analyses related to the lack of specifics regarding the distribution data/occurrence points. For example, based on the methods written on lines 209-211 and not being familiar with the AFE database, I thought that an AFE polygon meant there was one polygon for each species. But based on this, it is more clear that an AFE polygon is not related (maybe?) to the species themselves, but determined based on something else, and then the species occurrence data is mapped onto these polygons.

Lines 231-232: “We assigned the family name to each species to match the species distribution data with the available phylogenies.”

Why are family names important for the current study?

Lines 268-270: “To calculate environmental distances among data points, we extracted the values from the stack for all AFE points, standardized the bioclimatic values, and ran a PCA (princomp function in R) with all 19 standardized bioclim variables.”

What are the AFE points used in this analysis?

Line 293: Climate stability

I would like to know how the authors actually calculated stability, besides what R package was used (e.g. standard deviation)?

Line 300: Refugia section

The authors built SDMs to help identify areas of refugia; however, we are not given much information regarding their construction.

The authors calculated a GLM with only annual mean temperature and precipitation, why not use a standard algorithm with more variables?

What locality data was used?

Were there different levels of sampling bias for every species and was this accounted for in the models?

How was the performance of the SDM gauged?

Is there evidence these models performed well enough to project across time?

These models were only built using annual mean temperature and precipitation, do these two variables contain enough information to be used in these analyses?

Lines 325-327: “We calculated the mean range size among all species occurring in a sample location by taking the mean of the number of occupied AFE polygons across Europe per species present in any given sample location.”

Again, I am unsure what is meant by AFE polygons and the data they provide for the current analysis.

Line 341: Variable importance

It’s very surprising that only distance to refugia and climate stability were highly correlated because many of the response variables use the same underlying data (annual mean temperature and precipitation). It would be great for the authors to include the entire pairwise correlation matrix.

Minor typos:

Line 43, “climate change velocity”. Should this just be climate velocity? (and this phrase pops up elsewhere – lines 80-81, 83, for example).

Line 59, adaption --> adaptation

POINT-BY-POINT RESPONSE LETTER

Please find our point-by-point response to comments below. They are displayed in *italicized Arial font in blue* and examples of changed manuscript text are pasted in *"Times New Roman"* font and all changes made compared to the original text version are marked in *red*. The uploaded revised manuscript has all changes marked in red.

REVIEWERS' COMMENTS

Reviewer #1:
The paper deals with a very important topic and approaches this with an exciting approach using spatial phylogenetics and using a dated tree estimating plant movement and refugial areas. I am supportive of the work. But have many questions that are a result of some aspects of the manuscript that are poorly presented or unclear. Is this work on seed plants or all vascular plants? That is a big issue that needs to be clarified. I am also unclear on the phylogeny used and why - again was it all vascular plants or seed plants? The authors refer to both but most of the data seem to be angiosperms. The authors also need to clarify issues of the scale used (is this fine scale enough?) and the numbers of the specimens analyzed for each species (were enough locations used per species?). The work addresses a significant question and is highly relevant to present day as well as potential future plant distributions.

BS et al.: We highly appreciate the thorough and insightful comments by reviewer #1. We did our best to implement all concerns and suggestions of reviewer #1 and we are convinced that they improved the manuscript considerably. More specifically, we updated the manuscript as follows:

(1) Our analyses are on seed plants, namely angiosperms and gymnosperms, and not on all vascular plants. We realize that we were imprecise regarding this point and clarified this throughout the manuscript, notably on lines: 26, 72, 119, 153, 226, 253.

(2) There are many more angiosperms than gymnosperms in Europe – that's why there are many more species in the analysis on angiosperms than in the gymnosperm analysis.

(3) Scale: We used the quasi-rectangular 50 km grid, the original grain of the dataset, from Atlas Florae Europaeae (AFE) . It is the only quality-checked plant distribution data available that spans Europe. We were interested in broad trends in compositional change across Europe in our analysis of legacy effects from Quaternary climate cycles, and not small-scale variation in species composition. Nonetheless, we corrected for the effect of topographic variation in order to accommodate some smaller-scale variation in distributions especially in mountain areas. If we had finer scale data at hand, we would have aggregated it to this coarser resolution for exactly this reason of removing finer-scale variation. We therefore believe this is the proper scale for our analyses. This is now better explained in the manuscript (line 229ff, 304,407)

(4) Numbers of specimens: we realize that we were not clear enough in the methods section describing the AFE data set, which may have led to misunderstandings. We therefore clarified this in the methods section. AFE is a floristic mapping project, resulting in distribution maps of individual species at quasi-rectangular 50 km spacing. For each species used, we thus had the complete spatial distribution range available at this resolution, informing us about presence or absence within quasi-rectangular 50 km polygons. The AFE mapping is coordinated and synthesized in Finland, but all national floristic data authorities of Europe participate in this effort and quality-check each species' map before publishing. This is now better explained in the methods section (line 229ff).

23-24 we evaluate… phylogenetic diversity of European vascular plants…. Vascular plants are noted--But only angiosperms and gymnosperms are noted in the text

BS et al.: Indeed, this is correct. We used a wrong wording here as we only analyzed

angiosperms and gymnosperms, the seed and not the spore plants among the vascular plants. Thank you for careful reading and pointing this out. We changed it accordingly throughout the manuscript and replaced "vascular plants" with "seed plants".

Line 26, Introduction: "Here, we evaluate the spatial variation of phylogenetic diversity of European seed plants among neighboring sites and assess the effects of past rapid climate changes during the Quaternary on these patterns."

Line 72, Introduction: "Here, we quantify the phylo-β_{simC} of seed plants across Europe among neighboring cells."

Line 119, Results: "Southern Europe is characterized by a high species diversity of seed plants that persisted in refugia during the LGM (Fig. 4 and Supplementary Fig. 3)."

Line 153, Discussion: "In agreement with our general expectation, we find a clear spatial structure in phylogenetic turnover of seed plants across Europe, independent of regional environmental or topographic heterogeneity (phylo-β_{simC})."

Line 226, Material and methods: "Seed plant distribution data of angiosperms and gymnosperms were taken from the digital maps of the Atlas Florae Europaeae (AFE[57,58]) which includes ca. 25% of all European vascular plants."

Line 253, Material and methods: "Finally, we split the retained data into the two major clades, angiosperms and gymnosperms, and removed all other vascular plants."

24. The effect past climate change insert of. The effect of past climate change

BS et al.: Done.

Line 26, Introduction: "Here, we evaluate the spatial variation of phylogenetic diversity of European seed plants among neighboring sites and assess the effects of past rapid climate changes during the Quaternary on these patterns."

85. We analyze phylogenetic diversity separately for angiosperms and gymnosperms. What about other vascular plants (see above)?

BS et al.: As explained above, our primary goal was to analyze the influence of large-scale legacy effects from Quaternary climate cycles on regional species compositions across Europe. We used the only source of systematically sampled plant data for Europe, the Atlas Florae Europaeae. Analysis of the complete phylogeny of vascular plant species was not conducted because the presence or absence of single species of old clades could have distorted the result. We therefore calculated phylogenetic turnover for angiosperms (as a younger clade with many species, but incompletely sampled with regards to taxonomic coverage) and gymnosperms (an older, less specious clade that is sampled completely regarding taxonomic coverage) separately. We obtained the same pattern across both groups, which gives us confidence that the results are robust and unlikely to change much once the distribution of all angiosperm species is available. As a consequence of this sampling strategy, we did not analyze all vascular plants, as reviewer #1 correctly pointed out, only (vascular) seed plants as described above, we now adjusted the whole text accordingly. Including ancient and species poor clades such as Lycopsids, Filicales, etc. would have resulted in patterns of phylogenetic turnover driven by one or a few species potentially obscuring the more general patterns.

89-90 both angiosperms and gymnosperms... Again, what about other vascular plants?

BS et al.: See comment above.

150-151. Clear spatial structure in phylogenetic turnover of vascular plants ... But only angiosperms and gymnosperms are noted. What about ferns and lycophytes?

BS et al.: See comment above; we replaced "vascular plants" with "seed plants"

Line 153, Discussion: "In agreement with our general expectation, we find a clear spatial structure in phylogenetic turnover of seed plants across Europe, independent of regional environmental or topographic heterogeneity (phylo-β_{simC})."

161. Different mechanisms can explain...
Dispersibility ability is not ever discussed which seems surprising

BS et al. We thank the reviewer #1 for raising this point. We agree and find this point indeed important. We had only mentioned this point in the introduction (line 40-43) and discuss it now in the discussion section as well (line 164ff). Since dispersal ability is linked to the recolonization dynamics, we discuss the two elements together (see answer below and line 164ff).

179. Recolonization dynamics—. See comment above

BS et al.: We also followed this suggestion and added it to the discussion. The two elements of dispersal ability and resulting recolonization dynamics are now discussed in the discussion sections as follows:

Line 164, Discussion: "First, our results are in line with previous findings on the degree of range filling for European trees and plants[39-41]. These results indicate that many European plant species have not yet fully recolonized all suitable sites in Central and Northern Europe. Further, central and northern regions have been colonized by rather closely related generalist species with rapid dispersal capacities and, consequently, large ranges, while specialists generally have slower migration capacities and, thus, more restricted ranges[42]. Many of these specialists have not been able to migrate to these more distant regions that became ice-free around 10 millennia ago. Spatial turnover is thus directly linked to range size with smaller ranges enhancing turnover[43]."

195 projected future climate change is of equal if not larger intensity— refs?

BS et al.: We added a reference (Nr. 56), now line 213:

Line 568, References: "IPCC. Climate Change 2013: The physical science basis. Contribution of working group I to the fifth assessment report of the intergovernmental panel on climate change. (Cambridge University Press, Cambridge, UK and New York, NY, USA, 2013)."

200 the effect....have. Change to has

BS et al.: Done, now line 218.

208. Distribution data of angiosperms and gymnosperms... What about the other vascular plants? See comments above

BS et al.: See comment above.

220 Isoetes. These are lycophytes (not seed plants)— which again begs the question—what about these and other non-seed vascular plants?

BS et al.: We realize that we did not explain this clear enough, which has led to misunderstandings. The methods were written in the chronological order of data handling. We first cleaned the AFE dataset including all European vascular plants regarding their names using "The Plant List" and "Euro Plus Med Plantbase", including all vascular plants (that's why "Isoetes" is listed here). Only in a next step, we removed all species but angiosperms and gymnosperms. In order to avoid misunderstandings, we deleted the two non-seed species in this sentence and clearly state that we only kept angio- and gymnosperms in the data set and removed all other vascular plants. Thank you for carefully checking and spotting this issue.

Line 242, Material and Methods: "According to this, we kept five species (Cotoneaster

majoricensis, Malus crescimannoi, Papaver ernesti-mayeri, Pyrus castribonensis, Pyrus sicanorum) in our data as their names are accepted in E+M."

Line 252, Material and Methods: "Finally, we split the retained data into the two major clades, angiosperms and gymnosperms, and removed all other vascular plants. The angiosperm data set consists of 4003 species, 319 genera and 42 families, and the gymnosperm data set includes 41 species, 9 genera and 4 families."

226. Names. Again, what about non seed vascular plants?

BS et al.: Please see comments above.

238. Use of the Qian and Jin. See comment below

237-238. I think the most recent complete and updated tree is Smith and Brown 2018. The authors should note why Smith and Brown was not used? Also, it is a seed plant phylogeny as is Zanne et al. - I am not sure how non seed plants (the other vascular plants) were added here? This is a consistent problem in this ms.

BS et al.: Thank you for pointing us to the Smith and Brown phylogeny. We now removed the statement that the PhytoPhylo phylogeny is the "most up to date" phylogeny on seed plants (line 260) and we additionally ran all analyses using this alternative source of phylogenetic trees to test for robustness of our results. The results only differed marginally and did not change our findings and conclusions. We therefore kept the results from the Qian and Jin phylogenies in the main text and added the results based on the Smith and Brown phylogenies as a robustness test to the Supplementary (Supplementary Fig. 7). We further complemented the methods accordingly (line 260ff) and added a statement in the discussion (line 206ff) comparing the two results.

Line 260, Material and methods: "We used the species-level plant megaphylogeny (PhytoPhylo) of Qian and Jin[62] as a backbone tree and phylogenies of Smith and Brown[64] to test for robustness of our results (Supplementary Fig. 7)."

Line 206, Discussion: "Our results are robust regarding the use of different phylogenetic sources (Supplementary Fig. 7), and for both gymnosperms and angiosperms."

238. Qian and Jin is an update of Zanne et al — Zanne et al is a seed plant phylogeny and not for all vascular plants. How were other vascular plants added?

BS et al.: As explained above: we unfortunately used the wrong wording. We did not add any other vascular plants to the phylogeny. Only seed plants were used which we now make clear throughout the manuscript. See comment above to previous line 220 and the change in the manuscript that should make this clear now:

Line 252, Material and Methods: "Finally, we split the retained data into the two major clades, angiosperms and gymnosperms, and removed all other vascular plants. The angiosperm data set consists of 4003 species, 319 genera and 42 families, and the gymnosperm data set includes 41 species, 9 genera and 4 families."

241 Phytophylo includes all families of extant seed plants. What was done for non seed plants?

BS et al.: See comment above.

249. Total number of angiosperm species is given. We need totals for all vascular plants used in the study and how that compares to the actual total number for Europe

BS et al.: True, we agree that this is missing and we did not mean to hide this. Actually, the AFE database is not yet completely covering all angiosperm species of Europe. According to the AFE mapping committee, around one fourth of all vascular plants are available. 4664

angiosperm species and subspecies are currently available within the AFE dataset of all approximately 16'000 European angiosperms. On the other hand, the gymnosperms are complete. After collapsing subspecies to the species level, and after correcting names and merging the database with Phytophylo, we had 4003 species of angiosperm for analysis. As we get a very similar result for the complete gymnosperm dataset, we are confident that the angiosperm results are robust regarding this data bias. We now add the following statements:

Line 226 Material and Methods: "Seed plant distribution data of angiosperms and gymnosperms were taken from the digital maps of the Atlas Florae Europaeae (AFE[57,58]), which includes ca. 25% of all European vascular plants. The gymnosperms are complete, while the angiosperms are included to more than 25%."

Line 206, Discussion: "Our results are robust regarding the use of different phylogenetic sources (Supplementary Fig. 7), and for both gymnosperms and angiosperms. This consistency suggests that the same mechanisms likely drove these broad-scale biodiversity patterns across taxonomic groups in Europe. It further indicates that the angiosperm results are robust irrespective of analyzing only the mapped ca. 25% of European species."

268 resolution of 50 km. Other spatial phylogenetic studies are more fine grained than this. Why such a large scale?

BS et al.: This is the native resolution of the distribution data of the Atlas Florae Europaeae. We realize that our wording around using this atlas was not clear and concise. This floristic atlas does not present the distribution of plant species as range maps. Rather, the distribution is mapped across Europe in a quasi-rectangular 50 km spacing. In fact, they are mapped as polygons and they do not exactly represent 50x50 km raster cells. The species distribution data are then provided by the AFE committee as species lists per centroid of each of the >2000 quasi-rectangular polygons. For simplicity, we call these centroids "AFE points". We therefore have, for each species, its distribution available as a set of 1970 points within our study area with either presence or absence recorded at each AFE point. Distortions compared to a regular 50km lattice/raster are small but visible across Europe when transformed into a metric, equal-area projection. It is therefore not feasible to sample the data at finer spatial grains, as AFE does not provide this information. The quasi-rectangular 50 km distance among AFE points is the native resolution of the database. Rasterizing the data to a finer spatial grain would not solve the problem, as this would introduce a level of precision that is not real. We now provide a better description in the methods section, explaining the nature of the distribution data and the meaning of "polygons" and "centroid points" (see line 229ff) and we add information to the above cited reference of the 50 km resolution that this is the native resolution of the AFE distribution data (line 304) and on the focus on larger spatial trends.

Line 229, Material and Methods: "This atlas provides distribution data for each species mapped as presence or absence for each of quasi-rectangular polygons mimicking a 50x50 km raster across Europe. We extracted plant records within the extent of our study area, totaling to 1970 sample points representing centroids of the AFE distribution polygons (AFE points hereafter). Using these data, we generated community data of all species of gymnosperms and angiosperms present at these AFE points."

Line 304, Material and Methods: "We then re-projected and spatially aggregated the stack to the projection of the distribution files (LAEA) at a spatial resolution of 50 km, the native resolution of the AFE distribution data."

Line 407, Material and Methods: "[...] represents the larger scale variation across Europe that is independent of local topographic variation and is driven by other historical and contemporary drivers."

methods—how many specimens were examined per species in these inferences? Total number of specimens, etc?

BS et al.: We do not fully understand this comment, as we didn't use field-based specimens. But we try our best to discuss issues around sampling and species data.

We took distribution data from the digital distribution atlas of the European flora (AFE), meaning that we had – for each of the 1970 AFE points used (see map inserted in the answer to reviewer #2 under lines 209-211) – a full list of species available at each AFE sample point within our study area. Is this what reviewer #1 refers to as specimens? These data are quality checked by national mapping authorities (based on field data, photographs, herbarium records, etc.) before being released to the AFE atlas mapping team, which adds another round of quality checking. To be clear: we didn't remove any species from the database. If a species occurs only at 1 AFE point, it was included in the turnover analysis, because we do not make statements regarding single species. Rather we make statements regarding the community difference among AFE points. And in this analysis – as is standard in community ecology – we included all species irrespective of prevalence among AFE points. The fact that many species have restricted ranges (occur only in relatively few AFE points) then results in increased turnover rates (which is increased community difference). In order to avoid inserting an effect of richness difference between two communities in the quantification of turnover (community difference), we used the conservative "Simpson Index", which is unaffected by a possible richness difference among two compared communities.

We hope that our explanations have clarified the question. Of course, we are happy to change the text further, should reviewer #1 feel it is needed.

Reviewer #2:
I thoroughly enjoyed reviewing this paper, as it sheds light on an important topic in climate change research. Saladin et al. analyzed the effects of rapid climatic change during the Pleistocene on the phylogenetic diversity of vascular plants in Europe. The authors find that phylogenetic turnover is generally higher in Southern Europe (a region with low climate velocity) than Norther Europe, (a region strongly influenced by high climate velocity). The authors conclude the decrease in phylogenetic diversity across Northern Europe leads homogenization throughout the landscape due to unstable climates and distance from refugia. This manuscript is concise, well written, and provides an important contribution to the field. However, clarification on several key methodological points is needed, most notably the AFE database. Without this clarification of the analyses the paper is lacking. The points below could be considered to increase the impact of the paper.

BS et al.: Thank you for this positive feedback. We highly appreciate your careful and insightful review and we did our best to implement your suggestions. We are very happy about the points raised and we believe that the manuscript significantly gained in clarity and content. Specifically, we now better explain: (1) the species distribution data of the Atlas Florae Europaeae (AFE) and how it was used to generate community lists at a quasi-rectangular 50 km spacing, (2) the calculation of environmental distance among the 1970 sample points (AFE points) used in our phylogenetic turnover analysis, (3) the way how climate stability was calculated, (4) the model-based reconstruction of species' refugia using species distribution models and the KISSMig algorithm, and (5) the calculation of range size among European angiosperms and gymnosperms.

General comments:

Line 174 "Refugial areas can be expected in, or near, regions of high phylo-bsimC"

This is interesting wording since I would expect regions of high phylo-bsimC to be located mostly in areas of refugia. e.g., which is the driver of the relationship? Here, it's worded such that phylo-bsimC drives refugia, but in the introduction (lines 80-82), it's worded such that refugial dynamics drive phylo-bsimC.

BS et al.: We didn't intend to say that high turnover leads to refugial areas. Rather, we

wanted to express the idea that if we do not specifically know where to search for refugia, regions of high turnover (specifically North of the Alps) as were found in our study could be candidates because the presence of refugia may have led to the higher phylo-β_{simC}. So, we assume the same direction of the driver influence. We have now reworded the sentence in order to avoid confusion.

Line 183, Discussion: "Regions of high phylo-β_{simC} are candidates for Quaternary refugia."

Lines 186-192: High turnover in Northern and central locations

Do the authors have any insight in why these areas have high phylogenetic turnover? They mention these areas correspond to previous research, but is there anything they can add about these areas specifically regarding mechanisms that could produce the observed patterns? They do this nicely with low turnover in high climate velocity regions.

BS et al.: In the previous version, we did not speculate about the reasons behind these northern refugia. As expected by reviewer #2, we now add two possible explanations which we now discuss in the manuscript:

Line 201, Discussion: "Such refugia may have originated from locally more diverse environments, leading to a greater likelihood of refugial survival for some species in these heterogenous areas. Alternatively, higher phylo-β_{simC} in these refugial areas may simply arise from a lack of migration away from Quaternary refugia after climate warming, now leading to higher spatial turnover."

It would be interesting to see the stacked refugia maps for angiosperms and gymnosperms generated in this study and how they relate to the already known sites (R1, R2, and R3).

BS et al.: The stacked refugia maps are presented in Figure 4b for angiosperms and in the Supplementary Fig.3 for gymnosperms. Since the refugia are only hypotheses and not tested against found macro-remains or pollen data, we did not want to add direct comparison with species that add to turnover. It would also be difficult, because phylogenetic turnover can increase from several species being replaced, but also from few distantly related species being replaced. We therefore did not want to dig deeper into specifically analyzing the data at specific microrefugia (which often only are based on finding remains of one specific species).

Line 271: change sixth to six.

BS et al. : Done, now line 307.

Methodological comments:

Lines 209-211: "We included plant records within the extent of the study area, totaling to 1970 sample points representing centroids of AFE polygons."

Initial comment: I am quite confused about this - does this mean every species was represented by just one point at the center of their AFE polygon? If so, there seems to be a substantial mismatch between the number of species with distribution data and the number of species in the phylogeny. More detailed information is needed for the AFE polygons and what information they contain because I am not sure what is encompassed within this database.

Follow-up comment: Just before submitting the review, I noticed that the authors included a statement in their Nature Research "Reporting Summary" that "the presence and absence of each species is recorded for each of these sample points". Nothing like this statement was included in the main paper, and it would have been useful to head off a lot of confusion about the analyses related to the lack of specifics regarding the distribution data/occurrence points. For example, based on the methods written on lines 209-211 and not being familiar with the AFE database, I thought that an AFE polygon meant there was one polygon for each species. But based on this, it is more clear that an AFE polygon is not related (maybe?) to

the species themselves, but determined based on something else, and then the species occurrence data is mapped onto these polygons.

BS et al.: Please see also our comment to reviewer #1 for line 268 on the same issue of resolution of the distribution data. We realize that our wording around using the AFE atlas data was not concise enough to readers unfamiliar with the AFE data. This floristic atlas does not present the distribution of plant species as range maps. Rather, the distribution is mapped across Europe in ca. 50 km spacing. In fact, they are mapped as quasi-rectangular polygons and they do not exactly represent 50x50 km raster cells. The species distribution data are then provided by the AFE committee as species lists per centroid of each of the >2000 quasi-rectangular polygons. For simplicity, we call these centroids "AFE points". We therefore have, for each species, its distribution available as a set of 1970 points within our study area with either presence or absence recorded at each AFE point. The list of species present at each AFE point is thus the community list for our phylogenetic turnover analysis. As these centroids are ca. 50km apart, we refer to the sampling as a 50km grain. Distortions compared to a regular 50km lattice/raster are small but visible across Europe when transformed into a metric, equal-area projection. This means that we do not have overly simplistic data available but rather full (and quality-checked) species lists every 50 km across Europe. We now provide a better description in the methods section, explaining the nature of the distribution data and the meaning of "polygons" and "centroid points" (see line 229ff) and we add information under "Climate data" in same methods section mentioning that the 50 km resolution is the native resolution of the AFE distribution data (line 304).

Line 229, Material and methods: "This atlas provides distribution data for each species mapped as presence or absence for each of quasi-rectangular polygons mimicking a 50x50 km raster across Europe. We extracted plant records within the extent of our study area, totaling to 1970 sample points representing centroids of the AFE distribution polygons (AFE points hereafter). Using these data, we generated community data of all species of gymnosperms and angiosperms present at these AFE points."

Line 304, Material and methods: "We then re-projected and spatially aggregated the [climate data] stack to the projection of the distribution files (LAEA) at a spatial resolution of 50 km, the native resolution of the AFE distribution data."

For clarity, we add an illustration of the distribution structure of the Atlas Florae Europaeae, taken directly from the AFE website[1]. This map is larger than our study area, spanning considerably more than 2000 AFE points. See methods for explanation of confining the study area to a smaller extent:

[1] https://www.luomus.fi/en/new-grid-system-atlas-florae-europaeae

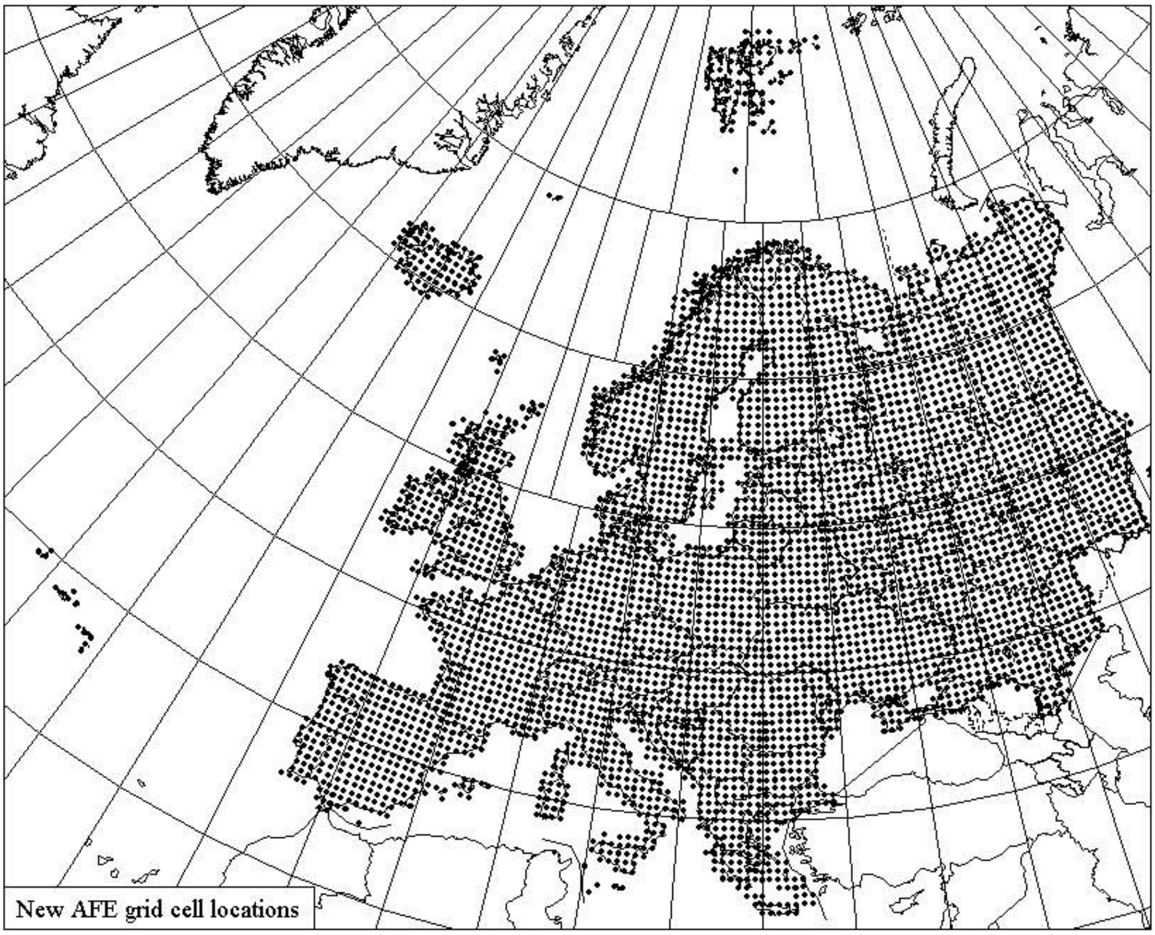

Lines 231-232: "We assigned the family name to each species to match the species distribution data with the available phylogenies."

Why are family names important for the current study?

BS et al.: We deleted this sentence as it causes more confusion rather than adding information. The sentence came from chronologically explaining elements of the data processing. When we checked for concordance in names between tips of the phylogenetic trees and the species included in the AFE dataset, 51 angiosperm genera (99 species) were not found in the phylogenetic trees. We removed them from the AFE data in our main analysis because they could not be unambiguously assigned to a synonym in the phylogenetic trees. Yet, as a robustness analysis, we also randomly added them as basal polytomies within the corresponding family (see Supplementary Fig. 8 for this). And that's why we had to assign the family name to each of these species. We therefore now delete the sentence in original lines 231-232. The rest is already described in the methods and this text should be sufficiently clear:

Line 267, Material and methods: While 51 angiosperm genera were not found in the PhytoPhylo tree, all gymnosperm genera were included. We removed the 99 species from the AFE data belonging to the 51 angiosperm genera not available in PhytoPhylo (see Supplementary Table 1 for the full lists) because they could not be unambiguously assigned to a synonym in the phylogenetic trees (see Supplementary Fig. 8 for results when including the species belonging to those genera by adding them as basal polytomies within the corresponding family).

Lines 268-270: "To calculate environmental distances among data points, we extracted the values from the stack for all AFE points, standardized the bioclimatic values, and ran a PCA (princomp function in R) with all 19 standardized bioclim variables."

What are the AFE points used in this analysis?

BS et al.: We assume that after changing the text relative to the comment on original lines 209-211 (now line 229ff) and the explanations given above, this becomes resolved. However, we realize that we also needed to be more explicit about the use of the AFE points in the distance calculation. To be clear, we repeat the explanation of what we did explicitly, and we refer for each aspect to the changed text.

We sampled each species across Europe at each of the 1970 AFE points where each point represents the centroid point of a quasi-rectangular polygon with ca. 50 km distance between polygon centroids (now explained in lines 229ff). We didn't rasterize the data, because the distribution data is not exactly aligned as a 50x50 km raster. At the same locations, we sampled the 19 bioclim variables at each AFE point location from the 50km bioclim grids (explained in line 304-306). Next, we ran a PCA on the 19 bioclim variables among all AFE points. Finally, we calculated the environmental distance among AFE points by means of the Euclidean distance calculated from the first six PCA axes (=environmental distance, now better described in lines 309-310). We hope that the procedure is now explained much clearer.

Line 229, Material and methods: "This atlas provides distribution data for each species mapped as presence or absence for each of quasi-rectangular polygons mimicking a 50x50 km raster across Europe. We extracted plant records within the extent of our study area, totaling to 1970 sample points representing centroids of the AFE distribution polygons (AFE points hereafter). Using these data, we generated community data of all species of gymnosperms and angiosperms present at these AFE points."

Line 302, Material and methods: "We then re-projected and spatially aggregated the [climate data] stack to the projection of the distribution files (LAEA) at a spatial resolution of 50 km, the native resolution of the AFE distribution data."

Line 309, Material and methods: "Finally, environmental distances among AFE points were calculated as the Euclidean distance using the first six PCA axis values."

Line 293: Climate stability

I would like to know how the authors actually calculated stability, besides what R package was used (e.g. standard deviation)?

BS et al.: The stability of climate is first calculated as "the mean of standard deviations between time slices divided by the time elapsed between time slices" (representing variability) separately for each of the two climate variables (here annual mean temperature and precipitation, using 1000-year time slices). Next, the inverse of these values (as stability = 1/variability) is taken per time slice to represent stability. The values are then re-scaled between 0 and 1 for each climate variable across Europe in order to allow comparison between variables. The overall climate stability is then generated by taking the product of the stability values from temperature and precipitation. The whole section describing climate stability computations is now much better explained in the methods section (line 343ff).

Line 343, Material and methods: "To measure climate stability, we applied the functions provided in the climateStability R-package[75]. We first measured the climate variability through time for annual mean temperature and annual precipitation sum using the same paleoclimate data with 1000-year time slices[74] as were used in climate change velocity calculations (described above). Variability was calculated as the climate difference between time slices expressed as standard deviation, and then the mean among all time slices was computed. We then inverted variability to stability (as stability = 1/variability) separately for temperature and precipitation, rescaled both variability maps between 0 and 1, combined the temperature and precipitation stability maps into a rescaled climate stability estimate by taking the product of the two maps, and rescaled the final map again to values ranging from 0 to 1, all following[75]. The final climate stability map was re-projected to LAEA at a 50 km resolution. This map indicates average stability of climate jointly for temperature and precipitation between LGM and present at a temporal resolution of 1000 years."

Line 300: Refugia section

The authors built SDMs to help identify areas of refugia; however, we are not given much information regarding their construction.

The authors calculated a GLM with only annual mean temperature and precipitation, why not use a standard algorithm with more variables?

What locality data was used?

Were there different levels of sampling bias for every species and was this accounted for in the models?

How was the performance of the SDM gauged?

Is there evidence these models performed well enough to project across time?

These models were only built using annual mean temperature and precipitation, do these two variables contain enough information to be used in these analyses?

BS et al.: We have improved the description of the elements mentioned above, and give now much more details on how the calculations were performed and how credible the results are. We refer to the different points mentioned above by underlining the main point raised by reviewer #2 first, and then give our response with associated text fragments where relevant.

BS et al.: Regarding the use of only temperature and precipitation – The SDM-based suitability maps used in the migration simulations to assess potential refugia cells are constructed from simple climate predictors, which one could consider too simple for modelling the current realized climate niche. For the dynamic modelling of past migration, however, robust hindcasting of potential suitability scores is important. Simple models have often proven to be more robust with projections than complex models and are especially advised to be preferred when projections to no-analogous conditions are envisioned (Merow et al. 2014), as is certainly the case here. The clear increase in model performance after adding simulated accessibility from the detected refugia demonstrates that the simple climate models are useful in combination with simulated migrations. This information has now been included (line 362ff and response on performance below). In addition, we explain that the dynamic reconstruction of the KISSMig algorithm additionally improves the reconstructions (line 384ff). In addition, more sophisticated past climate predictors were not available with the temporal resolution of this study.

Line 362, Material and methods: "The average performance of the initial GLMs evaluated by cross-validation and using the area under the ROC curve, AUC[76], was 0.89 ±0.083 for angiosperms and 0.88 ±0.069 for gymnosperms, indicating that these comparably simple models successfully fitted current plant distributions and are thus useful for hindcasting their distribution to the LGM."

Line 384, Material and methods: "The average deviance (D^2) explained increased from 0.48 to 0.68 for angiosperms, and from 0.44 to 0.67 for gymnosperms, respectively, after accounting for accessibility from refugial areas. The increase supports the adequacy of the model parametrization using annual temperature and precipitation for constraining the simulations of past migrations from candidate LGM refugial cells."

BS et al.: What locality data was used – We used the same AFE distribution data (1970 AFE points) for building SDMs as were used for the other analyses in the manuscript. This is now better reflected in the description of the methods (line 359).

Line 359, Material and methods: "First, we calibrated logistic regression models (GLM) for each species by using its current distribution points (AFE points) as response (prevalence = 0.5 by weighting) and current annual mean temperature and annual mean precipitation as climate predictors (entering the model as linear and quadratic terms)."

BS et al.: Regarding sampling bias – This is not easy to address as this information is not

available from the AFE consortium. However, we assume no clear or systematic difference in the sampling bias among the species mapped in AFE, as the mapping is performed in a very careful and time-consuming manner in which all countries are involved and maps are first prepared and quality-checked by each country representation, then given to the AFE office where the data are checked again, and then given back to country representations for final check before publication. This slow process is one of the main reasons, why since publishing the first volume of AFE in 1972, only ca. 25% of the European flora have been mapped.

BS et al.: On the SDM test performance – The SDMs performed well with an average cross-validated AUC value of 0.89 ±0.083 (s.d) for angiosperms and 0.88 ±0.069 for gymnosperms, indicating that the comparably simple SDMs generated useful models for migration assessments and identification of refugial cells. This information is now included and better described in the methods:

Line 362, Material and methods: "The average performance of the initial GLMs evaluated by cross-validation and using the area under the ROC curve, AUC[76], was 0.89 ±0.083 for angiosperms and 0.88 ±0.069 for gymnosperms, indicating that these comparably simple models successfully fitted current plant distributions and are thus useful for hindcasting their distribution to the LGM. For each species, we then projected its distribution under LGM climate and simulated migration from all suitable cells (i.e., suitablility > 0.5) separately for all 10 x 10 arcmin cells across Europe."

Line 618, References added: Zweig, M. H. & Campbell, G. Receiver-Operating Characteristic (Roc) Plots - a Fundamental Evaluation Tool in Clinical Medicine. Clin Chem 39, 561-577, (1993).

BS et al.: On evidence of SDM performance across time – The clear increase in model performance (D^2) for explaining the current species distribution (in the GLM) when adding accessibility from detected LGM refugial cells supports the usefulness of the climate predictors and initial SDMs for the simulation of migration through time. Would these models not provide an increase in D^2, there would be no evidence of added predictive performance. This information is now included in the description of the methods:

Line 384, Material and methods: "The average deviance (D^2) explained increased from 0.48 to 0.68 for angiosperms, and from 0.44 to 0.67 for gymnosperms, respectively, after accounting for accessibility from refugial areas. The increase supports the adequacy of the model parametrization using annual temperature and precipitation for constraining the simulations of past migrations from candidate refugial cells."

BS et al.: On the adequacy of using just temperature and precipitation for projections to past time slices – Temperature and precipitation alone may not contain enough information for projections of past species distributions using a static SDM. Species were not present at LGM in all areas of suitable climate, and this holds also if more sophisticated past climate predictors would have been available. However, in combination with the dynamic simulation of migration, temperature and precipitation contain enough information to generate meaningful potential LGM refugia by constraining their simulated migration. Moreover, the accessibility from refugia did not only improve SDMs under current conditions but showed also high variable importance in explaining the here analyzed patterns of spatial phylogenetic turnover. These two lines of evidence can be considered an adequacy test of the applied modelling approach. This information is now included in the description of the methods:

Line 386, Material and methods: "The increase supports the adequacy of the model parametrization using annual temperature and precipitation for constraining the simulations of past migrations from candidate LGM refugial cells."

Lines 325-327: "We calculated the mean range size among all species occurring in a sample location by taking the mean of the number of occupied AFE polygons across Europe per species present in any given sample location."

Again, I am unsure what is meant by AFE polygons and the data they provide for the current analysis.

BS et al.: We assume that this issue should now have become resolved. AFE polygons are equivalent to raster cells when rasterizing a distribution map. We have clarified this in the manuscript. So, we calculated range size as the number of polygons occupied by a species (equivalent to the number of AFE points occupied by a species).

*Line 393, Material and methods: "**Range size.** We calculated the mean range size among all species occurring in a sample location by taking the mean of the number of occupied AFE points across Europe per species present in any given sample location."*

Line 341: Variable importance

It's very surprising that only distance to refugia and climate stability were highly correlated because many of the response variables use the same underlying data (annual mean temperature and precipitation). It would be great for the authors to include the entire pairwise correlation matrix.

BS et al.: Good point, we now refer to the full correlation matrix among the spatial predictors of the residual phylo-β tested across Europe (line 409 & 414).

*Line 409, Material and methods: "**Variable importance and correlation.**"*

Line 414, Material and methods: "A full correlation matrix among the four predictors used is given in Supplementary Fig.12."

Minor typos:
Line 43, "climate change velocity". Should this just be climate velocity? (and this phrase pops up elsewhere – lines 80-81, 83, for example).

BS et al.: This term ("climate change velocity") has been used in the literature before (Ref 6 & 35) to explain the velocity at which climate is changing. As explained in the methods, we used the package "Vocc" which allowed us to calculate this metric. However, we realized that in Figure 3 we abbreviated "climate change velocity" to "climate velocity". We therefore replaced this abbreviation in Figure 3 and the Supplementary Figure 1 with those used elsewhere throughout the manuscript (DistRef, Vocc, RangeS). We adjusted the figure legends accordingly. In addition, we checked this term throughout the manuscript and changed "Climate velocity" to "Climate change velocity" in line 311, 337, 346. Thanks for spotting this.

Line 59, adaption --> adaptation

BS et al. : Changed, now line 61. Thank you for spotting this.

REVIEWERS' COMMENTS:

Reviewer #1 (Remarks to the Author):

The authors generally did a very good job of responding to the comments and concerns that I noted earlier. I think this version is much clearer and on point. It is well written and will be a valuable contribution that will receive a lot of attention once published

One semi-major point (perhaps—or maybe I am not understanding)—lines 210; 228-229 below. Plus a few minor things

87. Spell out angiosperms

121. Awkward sentence. While past climate change velocities were negatively, climate stability and species range size were positively related to phylo-bsimC (Fig. 3 and Supplementary Fig. 1).
Instead: While past climate change velocities were negatively related to phylo-bsimC, climate stability…

164-165. degree of range filling for European trees and plants…
Trees are plants! Reword

199. largely match with these hypothesized northern refugia ..
Reword
Largely matches the proposed locations of these hypothesized northern refugia …

209. taxonomic groups. Instead use "clades of life " this seems better

210. …only the mapped ca. 25% of European species.
So –75% missing taxa—that raises the question of biases in the species sampled…are some families underrepresented etc. See below. Some mention of this issue should be made—otherwise readers will wonder.

209-210. It further indicates that the angiosperm results are robust irrespective of analyzing only the mapped ca. 25% of European species.
I'm not sure how this totally shows robustness. You have only 25% of the angiosperms? You would have to see how results compared when you have a subclade (let us say a family) of angiosperms that is very well-sampled compared to subsamples of that family to say anything about "robust" results.

228-229. while the angiosperms are included to more than 25%. Exactly what does this mean? Only 25% are included? Does that mean a lot of missing data? How are these presence/absence data distributed across the phylogeny---for simplicity, across angiosperm families? What kinds of biases does this introduce if you have 75% missing? Are some lineages underrepresented relative to others that are overrepresented in that 25%? Please explain—or perhaps I am missing the point

288. Phylogenetic data. This section is a two page single paragraph. I would break it up into at least two. Perhaps line 288. Start a new para with "To test for robustness of our results …"

Reviewer #2 (Remarks to the Author):

I appreciate the hard work the authors have done to substantially improve their manuscript. I have a few minor comments for the authors to address before publication.

Minor comments:
Lines 171-172: “Spatial turnover is thus directly linked to range size with smaller ranges enhancing turnover.”

The text before this statement describes spatial turnover with regards to dispersal abilities, and I am confused about how this directly links to range size. I understand the correlations between generalist, better dispersal abilities, and larger range sizes but I am unsure about the “direct link” noted above.

Lines 209-210: “It further indicates that the angiosperm results are robust irrespective of analyzing only the mapped ca. 25% of European species.”

I would remove “the mapped” from this sentence.

Lines 228-229: “The gymnosperms are complete, while the angiosperms are included to more than 25%.”

I am unsure what is meant by this sentence.

Lines 229-231: “This atlas provides distribution data for each species mapped as presence or absence for each of quasi-rectangular polygons mimicking a 50x50 km raster across Europe.”

The grid cells of a 50x50 km raster are large. Do the authors think the grid size used here could affects their results? I think there should be a couple of sentences in the discussion about the size of grid cells used in the current study.

Line 231-232: “We extracted plant records within the extent of our study area, totaling to 1970 sample points representing centroids of the AFE distribution polygons (AFE points hereafter).”

Based on this and what is written in the response to reviewers: does this mean there are 1970 grid cells and you used the centroids of these cells to determine lat/long?

Figure 1: A map with some locations labeled would help readers understand what the authors are describing in the text for people unfamiliar with the area. This would also help with understanding Figures 3 and 4.

POINT-BY-POINT RESPONSE LETTER

Please find our point-by-point response to comments below. They are displayed in *italicized Arial font in blue* and examples of changed manuscript text are pasted in *"Times New Roman"* font and all changes made compared to the original text version are marked in *red*. The uploaded revised manuscript has all changes marked in track-change mode. Text *in green* represents sentences that have been moved to this new location (explained in our response from where it was moved to the new location).

REVIEWERS' COMMENTS

Reviewer #1 (Remarks to the Author):

The authors generally did a very good job of responding to the comments and concerns that I noted earlier. I think this version is much clearer and on point. It is well written and will be a valuable contribution that will receive a lot of attention once published

One semi-major point (perhaps—or maybe I am not understanding)—lines 210; 228-229 below. Plus a few minor things

BS et al.: Thanks a lot for your careful check on our revised manuscript and the positive feedback. We now provide more details in the text regarding the issues raised regarding lines 210, 228 and 229. Thank you!

87. Spell out angiosperms

BS et al.: Done (Line 87).

121. Awkward sentence. While past climate change velocities were negatively, climate stability and species range size were positively related to phylo-bsimC (Fig. 3 and Supplementary Fig. 1).
Instead: While past climate change velocities were negatively related to phylo-bsimC, climate stability…

BS et al.: We changed this sentence accordingly (Line 127). Thank you for your careful reading.

164-165. degree of range filling for European trees and plants…
Trees are plants! Reword

BS et al.: We deleted "trees and".

Line 154 Introduction: "First, our results are in line with previous findings on the degree of range filling for European plants[39-41]."

199. largely match with these hypothesized northern refugia ..
Reword
Largely matches the proposed locations of these hypothesized northern refugia …

BS et al.: Adjusted (Line 191).

209. taxonomic groups. Instead use “clades of life“ this seems better

BS et al.: Changed (Line 204).

210. …only the mapped ca. 25% of European species.
So –75% missing taxa—that raises the question of biases in the species sampled…are some families underrepresented etc. See below. Some mention of this issue should be made—otherwise readers will wonder.

BS et al.: Reviewer #1 is correct. The fact that all European gymnosperms are mapped in the AFE dataset, while only ca. 25% of European angiosperms are currently available as maps in the AFE database is one major reason why we split the analyses for angio- and gymnosperms. In fact, it didn't really make any difference: the older clade of gymnosperms essentially gave the same results as the selection of 25% of the younger angiosperm species (represented as selection of completely mapped angiosperm families, see new Supplementary Fig. 9). Because this selection of gymmo- and angiosperms provided the same results, we gained confidence that the missing 75% of angiosperms would likely not alter the results much. However, we now caution the “robustness” and refer to the fact that the results are only based on 25% of angiosperms (also in response to a comment by reviewer #1, see below re: line 209-210). With regards to completeness of angiosperm, please see also our response to reviewer #1 comment re: lines 228-229. All new text sections are explained in response to the next two comments by reviewer #1.

209-210. It further indicates that the angiosperm results are robust irrespective of analyzing only the mapped ca. 25% of European species.
I'm not sure how this totally shows robustness. You have only 25% of the angiosperms? You would have to see how results compared when you have a subclade (let us say a family) of angiosperms that is very well-sampled compared to subsamples of that family to say anything about “robust” results.

BS et al.: Thanks a lot for this comment. We now caution the “robustness”. We intended to say that the fact that the gymnosperms (available for and included to 100%) gave almost the same result as the available (and included) 25% of angiosperms indicates that the angiosperm results are robust. Yet, it is true, we cannot test or proof that without the remaining 75% angiosperms becoming available sometimes in the mid-term future (the AFE team will take several decades to complete the angiosperms for Europe). We therefore now caution this statement by indicating that it may indicate robustness, not that it does. In addition, we swapped the position of two sentences to be more logical in the argumentation. See also our response to the comment regarding lines 228-229.

Line 199, Discussion: “Our results are robust regarding the use of different phylogenetic sources (Supplementary Fig. 8), and for both gymnosperms and angiosperms. The fact that both gymnosperms and angiosperms showed very similar results suggests that the angiosperm results may be robust irrespective of analyzing only the currently available 25% of European angiosperm species. This consistency in our results suggests that the same mechanisms likely drove these broad-scale biodiversity patterns across clades of life in Europe.”

228-229. while the angiosperms are included to more than 25%. Exactly what does this mean? Only 25% are included? Does that mean a lot of missing data? How are these presence/absence data distributed across the phylogeny---for simplicity, across angiosperm families? What kinds of biases does this introduce if you have 75% missing? Are some lineages underrepresented relative to others that are

overrepresented in that 25%? Please explain—or perhaps I am missing the point

BS et al.: We changed this sentence also in response to reviewer #2, and we try to be more precise on our wording. The sentence means that while all European gymnosperms are mapped, only ca. 25% of all angiosperms are mapped and thus available for spatial analyses at the AFE resolution. With the new wording, this should now be clearer. In addition, we added a sentence referring to an additional supplementary figure (new Supplementary Fig. 9) illustrating what domains of the gymnosperm and angiosperm tree are covered by the mapped AFE data. Since the AFE team maps family by family, it is clear that many families are missing completely, while other families are included completely. Since this may question robustness of the results, we split the analyses into gymnosperms (maps available for all European species) and angiosperms (ca. 25% of species mapped). Since the results of the two very different groups (older gymnosperms vs. a selection of younger angiosperms) revealed very similar results, we think that a more complete angiosperm phylogeny would not result in strongly different patterns. The new text now reads as follows:

Line 235, Methods: "AFE maps are available for all European gymnosperms, while only ca. 25% of the angiosperms are mapped to date. The phylogenetic position of the available AFE angiosperm and gymnosperm data is illustrated in Supplementary Fig. 9."

288. Phylogenetic data. This section is a two page single paragraph. I would break it up into at least two. Perhaps line 288. Start a new para with "To test for robustness of our results …"

BS et al.: Thank you for your suggestion. We now start a new paragraph at the mentioned line (Line 303).

Reviewer #2 (Remarks to the Author):

I appreciate the hard work the authors have done to substantially improve their manuscript. I have a few minor comments for the authors to address before publication.

BS et al.: Thanks a lot for the positive feedback. We did our best to further improve the manuscript regarding the remaining minor points raised. Thank you!

Minor comments:

Lines 171-172: "Spatial turnover is thus directly linked to range size with smaller ranges enhancing turnover."

The text before this statement describes spatial turnover with regards to dispersal abilities, and I am confused about how this directly links to range size. I understand the correlations between generalist, better dispersal abilities, and larger range sizes but I am unsure about the "direct link" noted above.

BS et al.: Reviewer #2 is correct, there is no "direct" link. We simply remove the word "directly" from the sentence (Line 160).

Lines 209-210: "It further indicates that the angiosperm results are robust irrespective

of analyzing only the mapped ca. 25% of European species."
I would remove "the mapped" from this sentence.

BS et al.: Removed. This is a good point. In response to reviewer #1, we have adjusted this part anyway and have replaced the "mapped" by "currently available" (Line 202).

Lines 228-229: "The gymnosperms are complete, while the angiosperms are included to more than 25%."

I am unsure what is meant by this sentence.

BS et al.: Thanks a lot for this comment. We were likely not precise enough in our wording. We now extended this explanation, also in response to reviewer #1. The sentence meant to say that AFE maps are available for all European gymnosperms, while only ca. 25% of the angiosperms are mapped to date. AFE processes data family by family, thus many families are still unavailable within the AFE database. With the new wording, this should now be clearer.

Line 235, Methods: "AFE maps are available for all European gymnosperms, while only ca. 25% of the angiosperms are mapped to date."

Lines 229-231: "This atlas provides distribution data for each species mapped as presence or absence for each of quasi-rectangular polygons mimicking a 50x50 km raster across Europe."

The grid cells of a 50x50 km raster are large. Do the authors think the grid size used here could affects their results? I think there should be a couple of sentences in the discussion about the size of grid cells used in the current study.

BS et al.: This is an important issue, since phylo-β is scale-dependent. We have answered in the last round of revisions to a similar comment by reviewer #1, and we already made some adjustments to the text. Now we add a short section to the discussion to comply with the comment by reviewer #2. We argue that while it is the only data available for mapping plant species across all of Europe, it is essentially also a very appropriate resolution. We were interested in assessing broad spatial trends in regional compositional change across Europe in our analysis of legacy effects from Quaternary climate cycles, and not in small-scale variation in species compositions as can be analyzed at a plot scale. If we had finer scale data at hand, it would have been necessary to aggregate it to a coarser resolution for exactly the reason of removing finer-scale variation. Nonetheless, we corrected for the effect of local topographic variation on phylogenetic turnover (e.g. imposed by smaller-scale variation in distributions especially in mountain areas) in order to clearly emphasize the larger scale patterns that are independent of this local variation. If we had finer scale data at hand, we would have aggregated it to this coarser resolution for exactly this reason of removing finer-scale variation effects. We therefore believe this is the proper scale for our analyses. This is additionally discussed a

Line 204ff, Discussion: "Further, the analysis scale of 50 x 50 km in combination with removing local topographic effects on phylo-β_{simC} has proven to be appropriate for our analyses. Had we analyzed finer-scale data, local topographic and environmental effects might have been too dominant to discover larger-scale legacy effects (but see[56])."

Line 231-232: "We extracted plant records within the extent of our study area, totaling to 1970 sample points representing centroids of the AFE distribution polygons (AFE points hereafter)."

Based on this and what is written in the response to reviewers: does this mean there are 1970 grid cells and you used the centroids of these cells to determine lat/long?

BS et al.: Yes, correct.

Figure 1: A map with some locations labeled would help readers understand what the authors are describing in the text for people unfamiliar with the area. This would also help with understanding Figures 3 and 4.

BS et al.: We decided against adding this information to the Figure 1, as this would deviate the attention of the readers away from the main message (the resulting pattern) within this figure. Instead, we now additionally refer to the Supplementary Figure that illustrates the raw patterns at AFE locations (not interpolated across Europe to a 50 km raster) in the results section (Line 102). This figure was already contained in the supplementary material before but was cited much later. Now it is the first Supplementary Fig. 1, and readers can see a) how the AFE points are distributed, and b) how similar the raw and the interpolated patterns are. In addition, we also cite this new Supplementary Fig. 1 when introducing the AFE dataset in the methods section (Line 239).
